# A Review of Experimentally Informed Micromechanical Modeling of Nanoporous Metals: From Structural Descriptors to Predictive Structure–Property Relationships

**DOI:** 10.3390/ma13153307

**Published:** 2020-07-24

**Authors:** Claudia Richert, Norbert Huber

**Affiliations:** 1 Institute of Materials Research, Materials Mechanics, Helmholtz-Zentrum Geesthacht, 21502 Geesthacht, Germany; claudia.richert@hzg.de; 2 Institute of Materials Physics and Technology, Hamburg University of Technology, 21073 Hamburg, Germany

**Keywords:** nanotomography, 3D image processing, representative volume elements, geometrical characterization, descriptors, MD simulations, finite element simulations, deformation mechanisms, macroscopic mechanical properties, structure–properties relationships

## Abstract

Nanoporous metals made by dealloying take the form of macroscopic (mm- or cm-sized) porous bodies with a solid fraction of around 30%. The material exhibits a network structure of “ligaments” with an average ligament diameter that can be adjusted between 5 and 500 nm. Current research explores the use of nanoporous metals as functional materials with respect to electrochemical conversion and storage, bioanalytical and biomedical applications, and actuation and sensing. The mechanical behavior of the network structure provides the scope for fundamental research, particularly because of the high complexity originating from the randomness of the structure and the challenges arising from the nanosized ligaments, which can be accessed through an experiment only indirectly via the testing of the macroscopic properties. The strength of nanoscale ligaments increases systematically with decreasing size, and owing to the high surface-to-volume ratio their elastic and plastic properties can be additionally tuned by applying an electric potential. Therefore, nanoporous metals offer themselves as suitable model systems for exploring the structure–property relationships of complex interconnected microstructures as well as the basic mechanisms of the chemo-electro-mechanical coupling at interfaces. The micromechanical modeling of nanoporous metals is a rapidly growing field that strongly benefits from developments in computational methods, high-performance computing, and visualization techniques; it also benefits at the same time through advances in characterization techniques, including nanotomography, 3D image processing, and algorithms for geometrical and topological analysis. The review article collects articles on the structural characterization and micromechanical modeling of nanoporous metals and discusses the acquired understanding in the context of advancements in the experimental discipline. The concluding remarks are given in the form of a summary and an outline of future perspectives.

## 1. Introduction

Nanoporous metals made by de-alloying are receiving increasing attention due to their fascinating functional and mechanical properties, which can be controlled via electro-chemo-mechanical coupling through the huge surface inherent in this class of material. A series of reviews document the potential offered by the interface controlled functionality, particularly sensing and actuation [1], electrochemical conversion and storage [2], and bioanalytical and biomedical applications [3]. While originally, the research in this field concentrated on noble elements, such as gold, platinum, or palladium, the range of elements from which nanoporous materials can be produced by electrochemical dealloying has been substantially extended during the last few years with the help of liquid metal dealloying [4]. The different processes allow a design for solid fractions and ligament size as well as certain morphologies that can be modeled by kinetic Monte Carlo simulations or phase field modeling. A review of the characterization of the morphologies and mechanical properties can be found in [1,5,6].

Although the solid fraction and the average ligament size of mm-sized samples can be tuned to a desired value, the microstructure formed by the ligament network is highly random and, therefore, rather challenging for modeling. Lacking a detailed structural characterization, the interpretation of experiments is often based on the well-known Gibson–Ashby scaling laws [7], which represent a valuable and robust access to the mechanical properties of the solid phase. Under the assumption that the ligaments are sufficiently thin, the scaling of the macroscopically effective values of Young’s modulus E and yield stress $\sigma_{y}$ is given as function of the solid fraction φ as the following:

(1) $$\frac{E}{E_{s}}=C_{E}\varphi^{n_{E}}$$

(2) $$\frac{\sigma_{y}}{\sigma_{ys}}=C_{\sigma }\varphi^{n_{\sigma }}$$

where $E_{s}$ and $\sigma_{ys}$ denote Young’s modulus and yield stress of the solid phase, $C_{E}$ and $C_{\sigma }$ denote the leading constants, and $n_{E}$ and $n_{\sigma }$ denote the exponents. As summarized by Ashby and Bréchet, we have $n_{E}=2$ and $n_{\sigma }=3/2$ for bending dominated behavior, while for tension-dominated behavior $n_{E}=n_{\sigma }=1$ [8]. Gibson and Ashby pointed out that Equations (1) and (2) hold for low density cellular open foams—i.e., for solid fractions φ≤0.3 [9]. This range of solid fractions covers the majority of nanoporous metals reported in experimental works, but in some cases higher values have also been reported. Using Equations (1) and (2) for bending, which is the dominant deformation mechanism in foams and nanoporous metals deformed by macroscopic compression, the local Young’s modulus and yield strength within the nanoscaled ligaments is derived from nanoindentation experiments [10], macro compression tests [11], or micropillar tests [12], thereby consistently confirming the trend of “smaller is stronger” and approaching the theoretical yield strength of gold for ligament sizes from 100 down to 10 nm [12,13]. A significant amount of work went into the modification of the Gibson–Ashby scaling laws to integrate the phenomena observed in nanoporous metals. An overview of such modified scaling laws is given in [14]. 

Owning to the complex 3D structure, a more general description of nanoporous metals is challenging. Although high-resolution SEM images provide important insights into the details of the network structure, the generation of a 3D model requires assumptions that support the translation of this information into a realistic representation. Several approaches exist for generating random microstructures that are apparently very similar to nanoporous metals, but current research is trying to explore how to compare these microstructures. Therefore, an accurate geometrical characterization and structural description of the random network structure as a whole is important for two reasons: First, beyond the solid fraction φ and average ligament thickness d, which are usually provided in experimental works to characterize the microstructure, additional structural descriptors are needed to derive structure–properties relationships with enhanced generality and predictive capability; second, for producing reliable data, which helps to understand these structure–properties relationships, we need to ensure that real microstructures and computer-generated microstructures are comparable. Such comparisons are only possible once the relevant set of structural descriptors has been identified and can be automatically detected from experimental and computational 3D microstructures for a large number of features. Along this path, the obvious question is to determine how many nonlinearly independent structural descriptors exist and which of them are sufficient to uniquely define a microstructure as independent input to a structure–properties relationship. 

To give an example, the generation of random microstructures with the phase field method is not limited to low densities. Therefore, being unaware of the implications, there is a temptation to fit the simulation results for such structures with the Gibson–Ashby scaling laws. However, as clearly pointed out by Gibson and Ashby, the free length of the ligament becomes less than the ligament thickness for φ>0.3 and inaccuracies associated with the volumes of the nodes become severe [9]. Moreover, any form of the Gibson–Ashby model is based on a structure that has three ligaments connected in each node. Consequently, such scaling laws are only applicable if the coordination number of ligaments in the nodes remains unchanged (ideally three), while the solid fraction is varied. As soon as the solid fraction and the coordination vary simultaneously, a fit of the macroscopic properties versus the solid fraction leads to exponents in the Gibson–Ashby laws (1) and (2) that are usually higher than the ones derived by Gibson and Ashby, as for instance observed by Saane et al. [15]. This is just one example where additional information about the microstructure is needed to correctly attribute the predicted phenomena to the corresponding structural descriptors.

This review will have two foci. In Section 2 we will give an overview of the literature that deals with the characterization of the microstructure of nanoporous metals, thereby enhancing the knowledge relating to the description and quantitative comparison of the complex 3D network in terms of ligament geometry and network topology. For dealing with the rapidly increasing number of features and descriptors, the development of image processing software becomes a pressing issue. After relying on 2D SEM images for a long time, the pioneering work of Rösner et al. [16] on the high-resolution TEM tomography of np-Au represented a common starting point for in-depth 3D characterization, followed by X-ray tomography by Chen et al. [17]. It took about five more years until the first works on focused ion beam–scanning electron microscopy (FIB-SEM) tomography were published by Saane et al., Mangipudi et al., and Hu et al. [15,18,19], providing datasets for an advanced geometrical and micromechanical analysis. After this point, the number of publications in this field visibly increased.

The papers collected in Section 3 deal with the micromechanical modeling of nanoporous metals. Here the knowledge and quantitative description of the microstructure forms the basis for the setting up of micromechanical models—therefore, Section 2 and Section 3 are closely interlinked. In Section 3 we will distinguish two major approaches. The first is based on periodic and beam-like structures, taking up the developments of the foam community where the Gibson–Ashby scaling laws are still the most important. However, many other models have also been developed using unit cells such as gyroid, diamond, or cubic structures. Roberts and Garboczi extend this toward randomized networks by using Voronoi tessellations, but included also volumetric models like level-cut Gaussian random fields [20]. The latter represents the starting point of a second route, which is based on the phase field method or the kinetic Monte Carlo method, delivering random microstructures that show striking similarity to the real microstructures. 

A first overview emphasizing the role of computer simulations in the nanoporous metals community has been provided by Xia et al. [21], thereby summarizing relevant simulation methods concerned with dealloying and coarsening, as well as the mechanical and functional properties of nanoporous metals. Since then, research on the micromechanical modeling of nanoporous metals has been considerably advanced due to close links that have been established between modeling and experimental groups. Both areas challenged and inspired each other during the last two decades, and hence, a strong community has been formed and numerous developments on a fundamental level evolved. Therefore, it would fall short to reduce this review article to micromechanical modeling and instead we prefer the term “experimentally informed micromechanics” to emphasize the importance of this interdisciplinary collaboration. In view of what has been achieved, we hope with this review to inspire and motivate following generations of computational modelers and experimentalists to continue and further strengthen this valuable exchange.

## 2. Morphological Descriptors

Nanoporous metals exhibit a bi-continuous structure comprising the two phases—solid ligaments and pore space—with a characteristic feature size in the nanometer range and a solid fraction of around 30%. In its sponge-like appearance, nanoporous metals are similar to the well-studied material systems of open-cell foams and, furthermore, the trabecular bone. The main difference is the smallness of the feature size, and this makes the structural characterization even more challenging. Further, the morphology of nanoporous metals is even more complex. Owing to the randomness, distinct cage shapes, such as Kelvin or Voronoi cells known from the modeling of foams [20,22,23,24], cannot be easily identified. This comes along with the high requirements of a thorough and ideally automated structural characterization process. Pioneering work had been conducted from as early as 1992 and 2007–2010 [16,17,25,26], and these works are summarized as the starting point in the community in Section 2.1. 

**Figure 1:**
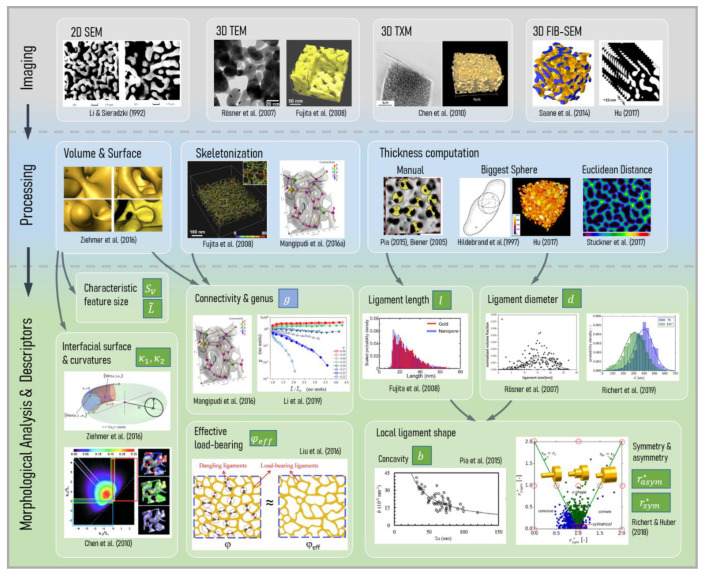
Overview of the morphological characterization divided into the three sequential stages of imaging, processing, and morphological analysis and descriptors. For figure reprint permissions, see Note at the end of the paper.

In this review we organize the full structural characterization process into three subsequent stages—the imaging techniques (Section 2.2), the image processing (Section 2.3), and morphological analysis with descriptor formulation (Section 2.4). These are depicted in the three rows of the overview shown in Figure 1, arranged from top to bottom. 

In Section 2.2 the relevant literature on the different imaging techniques for nanoporous metals is presented, as depicted in the first row in Figure 1**.** Scanning electron microscopy (SEM), transmission electron microscopy (TEM), transmission X-ray microscopy (TXM), and focused ion beam-scanning electron microscopy (FIB-SEM) are commonly used, but each has their own advantages and disadvantages. Nanoporous metals consist of two phases—the solid metal phase and the pore space—which need to be correctly separated. This binarization of the gray-scale image, namely segmentation, is a challenge on its own and the methodology depends on the imaging technique. 

At this point, a direct way to predict the mechanical behavior is to create a finite element (FE) model that is based on the experimentally informed solid phase volume. However, this will not answer the question of “why” because the image itself does not describe the structure in the sense of any mathematical abstraction. Subsequent image processing and characterization is required to examine the relationship between the geometric structure and mechanical properties. As an alternative approach, artificially created models resembling nanoporous metal structures allow the study of the influence of individual structural parameters on the macroscopic mechanical properties. However, these models need to be informed by the actual characteristics of the real microstructures to gain an understanding of a range of morphological parameters that are sufficiently close to reality. We divide the pre-stage of image processing (see Section 2.3) into three categories: (i) 3D solid volume confined by its surface, (ii) skeleton center-line, and (iii) feature thickness. For each task, different algorithms exist. Most of them are implemented in image-processing software packages, whose overview will also be given in connection with the relevant literature. 

Section 2.4 gives an overview of the different analysis methods and the descriptors that have been extracted from the surface, skeleton, and thickness information. Before we go into the details, it should be stressed that there exist different interpretations of the term “morphology” in the literature. In materials science, morphology is often used with respect to shapes or patterns, whereas topology means structure or connectedness of objects of arbitrary shapes. In engineering sciences, topology optimization has been established as a commonly used term for a methodology that is often limited to shape optimization, while it does not necessarily change a part in its topology. Ma et al. consistently use the mathematically correct terminology of “topology and shape optimization” [27]. Viergever et al. define mathematical morphology as “a theory for the analysis of spatial structures. It is called morphology since it aims at analyzing the shape and form of objects, and it is mathematical in the sense that the analysis is based on set theory, topology, lattice algebra, random functions, etc. Mathematical morphology is not only a theory, but also a powerful image analysis technique” [28]. As the structural characterization of nanoporous metals rests upon image processing and mathematical morphology, we will use the definition according to [28] in this review in the sense that morphology is the higher-level term integrating shape and topology. Consequently, morphology parameters can be divided into two subtopics called geometry, which studies the exact “shape”, and topology, which examines the “connectedness” [29] of a structure and does not consider lengths or angles.

For the geometric parameters, the solid volume allows the study of the solid fraction φ and the interfacial surface area A. Both parameters are of global nature and represent important independent parameters in terms of mechanical and functional properties. For a unique characterization, additional local measures are required. From the surface, local curvatures and surface normal orientations can be derived. Other geometrical parameters are the ligament length, tortuosity, or ligament thickness. The motivating insight behind topology is that some problems depend less on the exact geometric shape of the objects, but much more on the way they are put together. In a nutshell, topology parameters, such as connectivity or genus, are related to the number of continuous “handles,” “tunnels,” or “loops” in the structure. 

For a bicontiuous structure, all parameters discussed in the following can be calculated for the solid phase as well as the pore space. For the sake of simplicity, we limit our perspective to the solid phase controlling the mechanical behavior, whereas the pore space would be of primary interest for fluid flow and diffusion processes responsible for many functional properties. Therefore, only parameters regarding the solid phase are shown in Figure 1.

### 2.1. Pioneering Work

In 1992 Li and Sieradzki reported the first characterizations and mechanical fracture properties of 3D random porous gold [25], which to that point had been studied by simplification to 2D model structures. For characterization, the morphology of the electrochemically leached-out parent alloy (${\mathrm{Ag}}_{0.76}{\mathrm{Au}}_{0.24}$) is described as an “interpenetrating solid-void composite similar to the structure of porous Vycor” [25]. As an imaging technique, 2D SEM was used for the coarsened structures, while scanning tunneling microscopy (STM) and transmission electron microscopy (TEM) are used to examine the dealloyed sample. The as-dealloyed structures showed a feature size of about 3 nm. Subsequent coarsening treatments resulted in structures with feature sizes by more than two orders of magnitude larger at the highest temperature of 800 °C. The authors conducted three-point bending experiments and observed a ductile to brittle transition in the mechanical properties, where samples annealed above 300 °C developed ductile properties. 

Corcoran et al. used in-situ small-angle neutron scattering (SANS) measurements to analyze the structure formation of nanoporous gold during free corrosion and dealloying under the potential control of a ${\mathrm{Ag}}_{0.7}{\mathrm{Au}}_{0.3}$ parent-alloy [30,31]. They characterized the evolution of the distribution of the average ligament diameter as a function of the coarsening time ranging from a few minutes up to 21 h. For the free corrosion, a peak in the SANS data is observed already after a few minutes. With increasing time, the peak shifted to smaller scattering vectors Q, which corresponds to an increase in the average ligament diameter. Normalizing the data showed an overlap of the curves, which indicates self-similar coarsening of the porosity [30]. A different behavior was observed for the potential controlled dealloying. No coarsening of the porosity was observed at an applied potential of 700 mV, which was above the critical dealloying potential for the system. The absolute scattering data showed a broad peak with weak intensity even after 15 h of the coarsening time. After lowering the potential to 450 mV, a peak became visible and the coarsening took place. To determine the ligament diameter distribution from the absolute scattering data, the authors created model structures following the method by Berk [32]. To mathematically model the bicontinuous pore morphology, stochastically generated standing leveled waves were superimposed. This led to a 3D model that was used to analyze the average ligament diameter distribution. For structures undergoing coarsening via surface diffusion or via bulk diffusion, the ligament diameter d increases proportionally to $t^{1/4}$ or $t^{1/3}$, respectively, according to the well-known power law $d=t^{1/n}$. Applying the power law $d=t^{1/n}$ to the ligament diameter d of the free-coarsened sample as a function of time t revealed a change of the exponent 1/n from 1/4 to 1/3, i.e., from surface to bulk diffusion, after 4 h. For the sample made by potential controlled dealloying, a constant exponent of 1/3 (0.28) was observed. 

Rösner et al. have established pioneering work in the characterization of the 3D microstructure by analyzing a TEM tomographic reconstruction of a nanoporous gold leaf fabricated by dealloying (${\mathrm{Ag}}_{80}{\mathrm{Au}}_{20}$ alloy) [16]. They analyzed microstructural parameters such as the volume fraction of the two phases, ligament and pore size distribution, specific surface area, surface orientation distribution, and the mean curvature. To analyze the complex topology and branch lengths, the solid and pore phase were each skeletonized and “dead ends” of ligaments, which are only one-side-connected ligaments, were observed. For further characterization, a stereological analysis was performed using the software MAVI—Modular Algorithms for Volume Images by Fraunhofer. From granulometry and volume-weighted local feature size, the authors highlight a broad distribution of ligament shapes and diameters ranging from 5 to 30 nm. They also point out the structural diversity that “small pores and small, almost circular ligament rings coexist with much larger and quite complex pores and rings” [16]. Further, ligaments with an elliptical cross-section are found. With this comprehensive work on such a variety of morphological parameters, Rösner et al. have contributed tremendously to establishing feature descriptions, as the authors optimistically stated in their conclusion. Looking back, it can be confirmed that they have set a standard and inspired the growing community at an early stage of research.

Fujita et al. have analyzed a nanoporous gold film made by dealloying (alloy ${\mathrm{Au}}_{35}{\mathrm{Ag}}_{65}$) using TEM imaging and 3D reconstruction [26]. The resulting solid fraction was measured as approximately 0.5. Their curvature analysis indicates a net-zero curvature surface. The Fourier transform revealed a characteristic length scale of 30 nm, which they associate with a statistical period length of the structure. A skeleton was calculated using surface thinning to measure the ligament and the pore canal length, which showed asymmetric distributions. The diameters of gold ligaments and pores were determined by counting the removed pixels from the surface to the center of the ligaments on the skeleton network. The diameter distributions were found to follow a Gaussian-like distribution. For the investigated solid fraction of φ=0.5, the ligaments and pore canals seemed to be geometrically identical—this is in contrast to the results of [16], which had a lower solid fraction. Fujita et al. conclude that the morphology strongly depends on the parent-alloy composition and also the dealloying conditions [26].

Chen et al. were the first to use transmission X-ray microscopy (TXM) with 30 nm resolution and tomographic reconstruction on a coarsened np-Au wire (Ag-30 at.% Au alloy) with an as-dealloyed gold solid fraction of φ=0.5 [17]. Their analysis results show an interfacial shape distribution (ISD) of a near zero average mean curvature (see Figure 5) in agreement with the result of Fujita et al. [26], as both studies examined np-Au samples with a solid fraction φ=0.5. Further, the authors state that local positive Gaussian curvatures resemble pinch-off events, which indicate a local transport mechanism like surface diffusion but not bulk diffusion (general information about the curvatures and corresponding variables can be found in [5], see Section 2.4.2). Concerning the local ligament shape, the so-called cylindrical regions with zero Gaussian curvature are described as transitions between patches with positive and negative mean curvatures. Besides the interface shape, they analyze the topological characteristics in more detail by determining the genus g by integrating the Gaussian curvature. In a subsequent study, Chen-Wiegart et al. have analyzed the three-dimensional evolution of the morphology during isothermal coarsening (free corrosion) using ex-situ TXM measurements [33]. They report no significant densification during the coarsening at a solid fraction of approximately φ=0.28. However, they find a broadening of the interfacial normal distributions (ISD) plots at later evolution steps, thereby indicating a non-self-similar coarsening behavior. Further, their feature size evolution does not fit the well-known power law $d=t^{1/n}$ of a coarsening process, which underlines the hypothesis of non-self-similar coarsening behavior for their experiment [33]. 

The pioneering works summarized in this section introduced different imaging techniques as well as image-processing methods for nanoporous metals. They have laid the foundation for the morphological characterization and analyzed a number of important measures for the description of the shape and topology of the microstructure. In what follows, the literature is reviewed in terms of further development. New insights have been obtained and grouped by the three main subsequent stages—imaging techniques (Section 2.2), data processing (Section 2.3), and morphological analysis with descriptor formulation (Section 2.4). A detailed review of the experimental methods and imaging techniques is beyond the scope of this work. Our focus is set on Section 2.3 and Section 2.4, as these contents strongly influence the research in the field of micromechanical modeling and numerical simulation. 

### 2.2. Imaging Techniques

During the last two decades, the structural characterization and prediction of mechanical properties of open-pore nanoporous metals gained increasing attention. Benefitted by the improving resolution of imaging techniques, this was complemented by advances in image processing algorithms and computational modeling techniques. For the morphological analysis, 2D structure information from cross-sections can provide valuable insights, but especially, the analysis of topological parameters calls for 3D image reconstructions and image processing.

**Figure 2:**
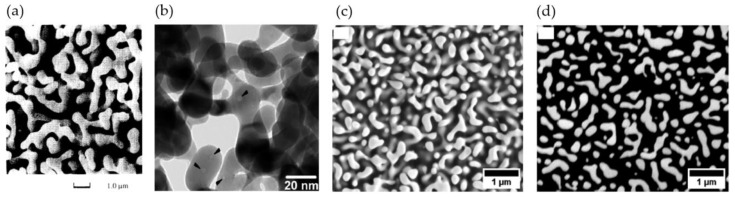
Nanoporous gold imaged using (**a**) 2D SEM by Li and Sieradzki [25]; (**b**) TEM by Rösner et al. [16]; (**c**) SEM image of a focused ion beam (FIB) milled cross section of epoxy infiltrated nanoporous gold showing shine-through effect of underlying structure by Hu [36] and (**d**) effect eliminated by choosing different electron beam voltage [36]. Reprinted with permission from [25] Copyright (2020) by the American Physical Society; [16] with permission from John Wiley and Sons; [36] with permission from Kaixiong Hu.

As described in more detail in Section 2.1, Li and Sieradzki presented the first scanning electron microscopy (SEM) images of nanoporous gold (np-Au) at successive coarsening stages [25], as seen in Figure 2a. The first 3D studies based on np-Au were conducted by Rösner et al. using the transmission electron microscopy (TEM) measurements of nanoporous gold leafs [16], see Figure 2b. Fujita et al. have analyzed a nanoporous gold film using TEM and 3D reconstruction [26]. TEM has a high spatial resolution, but it is limited to the analysis of thin films. Chen et al. have used transmission X-ray microscopy (TXM) on a coarsened np-Au wire [17]. As a more recent development, Mangipudi et al. [34] and Hu et al. [19] have simultaneously introduced the analysis of np-Au samples of much larger sample volumes by using a combination of destructive focused ion beam (FIB) sectioning and scanning electron microscope (SEM) imaging, see Figure 2c. Zabihzadeh et al. have performed ptychographic X-ray computed tomography (PXCT) on nanoporous silver laminate films, with resulting 3D resolution ranging from 35 to 55 nm but of a limited sample size [35]. 

Across all imaging techniques, challenging tasks include beam alignment, layer thickness, surface evenness, and view of depth. Depending on the imaging technique, the 3D structural data needs to be reconstructed from a 2D image series first, such as in X-ray TXM or electron tomography (ET). In ET, the 2-D projection images taken at different viewing directions in a tilt series are used for reconstructing the 3D image [37]. This reconstruction can be computed using different algorithms such as Fourier reconstruction, weighted back-projection (WBPJ), or iterative reconstruction algorithm [38,39]. Other algorithms, such as total variation minimization (TVM) and discrete algebraic reconstruction (DART), are compared in [40]. Machine learning approaches for reconstruction are advancing fast these days. A broad overview is given in [41] and the related articles.

In FIB-SEM serial sectioning, the 2D images resemble physical cross-sections through the structure. As the process of sectioning and imaging is repeated until the desired volume is scanned, the required imaging time increases with the sample volume and the resolution, yielding durations of several hours up to days. The 3D reconstruction is not trivial, as in porous structures the features below the surface are visible through the pores; the so-called shine-through effect as can be seen in Figure 2a,c. Care needs to be taken because this can lead to substantial errors in the thresholding and later volume reconstruction. Hu et al. and Mangipudi et al. have minimized this effect by infiltrating the sample with either epoxy or an electron beam-opaque Pt-based nanocomposite, respectively [18,19]. Mangipudi et al. have introduced a wedge-shaped sample allowing for an accurate calculation of the actual slice thickness [18]. In traditional serial sectioning, mostly rectangular Volumes of Interest (VOI) are scanned and a constant slice thickness is assumed. The authors show a visible influence of the correct slice thickness estimation on the morphological and topological features, which are ligament and pore size distributions, interface shape distribution functions, interface normal distributions, and genus. Further, re-deposition during milling, edge effects, and non-flatness of the milled section can affect the imaging quality.

In conclusion, each imaging technique has its advantages and disadvantages. High spatial resolution often comes along with limitations in sample size or increased imaging time. Many influencing factors need to be treated carefully, depending on the imaging technique. After the image collection, the image processing as the subsequent step holds further challenges.

### 2.3. Image Processing

For the image processing of nanoporous structures, we address in this review the three categories 3D volume/interfacial surface (VS), skeleton centerline (SC), and feature thickness (FT). After the image processing, the workflow extends to the analysis and computation of geometrical and topological structure features and descriptors—i.e., the morphological analysis and descriptors (MA), see following Section 2.4. In recent years, the community established and adopted a growing number of open-source software (OSS) and commercial software packages for the image-processing and morphological analysis. An overview of the most commonly used software is given in Table 1 and arranged in alphabetical order. The functionality in the main processing categories (VS, SC, FT) as well as the morphological analysis (MA) is marked with X (yes) or—(no). 

#### 2.3.1. Segmentation and Surface Extraction

All imaging techniques have the need for segmentation in common—i.e., binarization of the grey-scale image data—to distinguish between solid gold and pore space. This is done by setting a threshold value, which categorizes the grey-scale pixel information into black and white (on/off) pixels. For a detailed structural and mechanical analysis, it is critical to reproduce the 3D surface as closely to the originally structure as possible. According to Equation (1), for a typical solid fraction φ≈0.3, an error of only 10% in the solid fraction propagates into an error of 20% in the macroscopic Young’s modulus. Therefore, even for a robust measure as the solid fraction φ, an accurate reconstruction of the microstructure from the grey-scaled images is essential. 

Seker et al. discuss in their review the difficulties involved with the segmentation process based on mainly 2D SEM images [56]. Prior to segmentation, data smoothening can be conducted to reduce the level of noise by, for example, standard 3D Gaussian smoothing [33]. A common way is to fit the threshold such that the solid fraction of the reconstructed structure fits the solid fraction of the experiment [16]. For this, it is important to consider the volume shrinkage of the sample during dealloying [57]. A common thresholding algorithm has been proposed by Otsu [58], which is implemented in MATLAB’s internal function “graythresh” [34] and in Fiji/ImageJ [48]. Other thresholding algorithms, such as Huang, Intermodes, IsoData, Li, MaxEntropy, Mean, MinError, Minimum, Moments, Percentile, RenyiEntropy, Shanbhag, Triangle, and Yen, are implemented in Fiji/ImageJ [48]. 

For most subsequent analyzing algorithms, the reconstructed binarized image data of the structure should be fully interconnected. Thus, all free-floating segments originating from the cut at the boundary should be removed [16]. This operation is called “purify” in the community [26] and, for example, is available in the open-source software Fiji/ImageJ [48]. As a first attempt, the voxelized structure can be used for analysis of the interfacial surface area, but it is more accurate to mesh the voxelized binary structure. Mostly standard triangulation language (STL) is used for that and it can be achieved using Aviso-Amira (ThermoFisher Scientific) or the “iso2meshtoolbox” [51] as used in [42] or [18,34], respectively.

#### 2.3.2. Skeletonization

**Figure 3:**
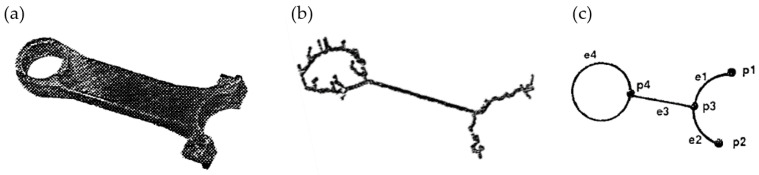
Skeletonization process schematic on an example of a connecting rod: (**a**) 3D model, (**b**) error skeleton spurs due to surface roughness, (**c**) idealized graph theory representation of edges “e” that are connected in vertices “p”. Reprinted from [60] with permission from Elsevier.

The skeleton—i.e., the medial axis—is the one-voxel-wide centerline that preserves the topology of the solid phase and is computed by means of the hit-or-miss transform [59]. The most frequently used skeletonization approach by Lee et al. applies surface thinning, where the surface voxels are iteratively eroded until only the centerline remains [60]. An alternative approach is to calculate the distance transform of the image where the skeleton lies along the local maxima of the distance transform [60,61]. The commonly used skeletonization codes implemented in automated imaging software programs are “Skeletonize3D” in Fiji/ImageJ based on the surface thinning algorithm by Lee et al. [60], the plugin “AnalyzeSkeleton” [62], or “Bwmorph” in Matlab. The resulting one-voxel-wide skeleton network is made up by interconnected so-called nodes and ligaments, or junctions and branches, or vertices and edges. Such an idealized skeleton of a 3D model of a connecting rod, see Figure 3a, can be expressed as an un-weighted network according to graph theory, see Figure 3c [60].

Skeletonization is very sensitive to the surface roughness, which is mainly related to the voxel resolution. Two options are possible to deal with this, referring to [60]: “It is possible that few spurs still remain in the skeleton because of the small variations in the surfaces of the object. In this case, we can either use a pre-processor such as a 3-D digital filter to smooth out noises in 3-D digital images before applying the thinning process or use a post-processor to remove spurs in the skeleton afterwards by thresholding on the length of each skeleton arc.” Such skeleton spurs are depicted in Figure 3b, resulting from the voxel representation with digitization noise applied to the designed connecting rod in Figure 3a [60]. Most skeletonization toolboxes, therefore, have the possibility of “edge pruning,” where a threshold can be specified for short and only one-side connected ligaments to be deleted.

Besides the challenge of reflecting the true geometric features while noisy surfaces exist, difficulties with the skeletonization of nanoporous structures can occur at the boundary of the Region of Interest (ROI). The skeleton axis will not end exactly on the boundary plane, but rather further inside the ROI volume. With this, one does lose information about whether the structure was interconnected across the boundary or not [19,50]. 

#### 2.3.3. Thickness Computation

**Figure 4:**
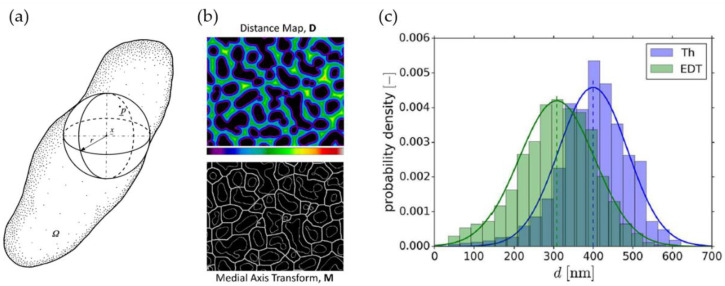
Schematic of thickness estimation algorithms (**a**) biggest-sphere Thickness (Th) by Hildebrand and Rüegsegger [63], (**b**) Euclidean Distance Map (EDT) and multiplication with the Medial Axis Transform (MAT) skeleton used by Stuckner et al. [44]. (**c**) Significant shift in the diameter distribution is observed using the Th and EDT algorithm on one tomography: average diameter ${\langle d\rangle }_{Th}=400$ nm and ${\langle d\rangle }_{EDT}=308$ nm by Richert et al. [53]. Reprinted from [63] with permission from John Wiley and Sons; [44] with permission from Elsevier; the author’s own work [53] published under Creative Commons Attribution License (CC BY 4.0).

The measurement of the thickness in a 3D volume is not possible by manual means. To have more reliable and faster results, automated image processing software is commonly used. Different algorithms for the estimation of the ligament thickness of nanoporous structures can be found in the literature. Rösner et al. have quantified the pore and ligament sizes using an approach that comes from granulometry where the pore or ligament space is completely filled by overlapping spheres of different sizes. Next, each voxel in the phase is assigned to the largest sphere in which it was contained [16]. This resembles the biggest-sphere Thickness algorithm by Hildebrand and Rüegsegger, see Figure 4a [63], which is implemented in image processing software, such as the open-source program Fiji developed by Schindelin et al. [48] in the BoneJ Plugin [64]. It calculates the local thickness at a point as the diameter of the largest sphere, which is completely inside the structure and which contains the evaluated point. By this definition, the biggest sphere at a skeleton point does not need to be centered at this point. Further, the mean thickness is calculated as the volume-weighted average of the local thickness of all voxels. In the context of np-Au, the biggest-sphere Thickness algorithm has been applied for analyzing 3D tomography data or voxel models by [18,19,43,44,65] for analyzing their samples with respect to the thickness distribution. 

In recent years, using the Euclidean distance transform (EDT) of the structure volume has received more attention. The EDT determines the distance to the nearest pore-space voxel at each voxel of the solid phase. Concerning the average ligament thickness and the thickness distribution, only the distance values along the skeleton line shall be evaluated—this can be achieved using an image multiplication with the skeleton. Without this voxel reduction, a volume-based distribution would be biased toward smaller values that originate from the voxels that are located outside the skeleton line and are, therefore, closer to the pore space. Similarly, Fujita et al. have already calculated a skeleton using surface thinning and determined the ligament diameters by counting the removed pixels from the surface to the center of the ligaments on the skeleton network [26]. The Euclidean distance transform (EDT), in combination with the skeleton line as an image multiplication, was applied to 2D SEM images by Badwe et al. [49] and implemented by Stuckner et al. in their software package AQUAMI [44], which was used in the data mining study of McCue et al. [45]. Richert et al. have applied the EDT in [53] in combination with skeletonization to the 3D tomography dataset from [19].

Richert et al. have found that for nanoporous gold, the biggest-sphere Thickness algorithm systematically overpredicts the local thickness assigned to a certain skeleton voxel by up to 30%, which can lead to overprediction in the macroscopic stiffness and strength by up to 100% for concave ligament shapes [50,53]. This effect is pronounced for shapes, which have a strong evolution of local diameters along their axis. It is shown that the biggest-sphere Thickness algorithm tends to smear the local thinning by propagating the ligament thickness from the thicker into the thinner parts. The larger the thickness gradients, the larger the effect. In comparison, Richert et al. have used the 3D EDT algorithm in the TANGO plugin [66] in Fiji to characterize a 3D FIB-SEM tomography by Hu et al. [19]. In comparing this method to the biggest-sphere Thickness results, the determined diameter distributions are shifted and the averages deviate significantly, being 400 nm (Th) and 308 nm (EDT). 

Using artificially created model structures, Richert et al. could show that the EDT algorithm delivers much better accuracy compared to the biggest-sphere Thickness algorithm. Although it systematically underestimates the local thickness, the error is much lower, thereby limiting the underprediction in the macroscopic Young’s modulus to 20% [53]. The reason for the underprediction is as follows: the distance is determined by counting voxels from the surface contour normal to an axis point (attributed to the axis point). This value is equal or smaller than the value measured, starting at that axis point and counting along the axis normal to the surface. 

In their conclusions, Richert et al. point out that the results of the EDT algorithm should be critically evaluated with respect to effects from non-circular cross-sections that might occur in real samples. This could, for instance, be measured using the star-line-based algorithm proposed by Liu et al., where the thickness at an axial voxel is defined as the minimum-intercept of a straight line with the interfacial boundary [67]. One drawback of this thickness computation method lies in the increased computation time needed. Although it is discussed and used in the medicine community [68,69,70], this algorithm is yet to be used by research groups in the community of nanoporous metals.

Since the mechanical properties are very sensitive to the ligament size, care needs to be taken in calculating the local diameter. As we have learned, the EDT slightly underestimates the true thickness, whereas the Biggest-Sphere algorithm leads to a significant and systematic overestimation. This is not just important to keep in mind while comparing the thickness data gained by different authors, who probably applied different thickness estimation algorithms. Moreover, a correct estimation of local diameter values along the ligament axis is essential for the quality of predictions using FE methods. 

### 2.4. Morphological Analysis and Descriptors

The progress regarding the structural characterization and evaluation of useful descriptors is presented in the following. Much can be learnt from the materials science community with regard to the meaning and use of these measures for addressing particular scientific questions. This forms a valuable foundation for the development of micromechanical models and the interpretation of simulation results. Therefore, we pay particular attention on how this knowledge can be incorporated in micromechanical modeling. While the modeling community grows stronger, we can observe a trend where descriptors are being developed driven by the needs of continuous improvement of the micromechanical models—this leads to a close interaction between both communities. 

For a review of the principles and different processing routes of electrochemical deallyoing of metals, the reader is referred to [71]. To study the influence of the ligament size on the yield strength, McCue et al. have collected data from several other research groups of np metals such as np-Au, np-Cu, np-Pd, and np-Ti. In their following review [4], McCue et al. discuss the evolving process of liquid metal dealloying (LMD) in comparison with the classical electrochemical dealloying (ECD) regarding the pattern formation, with both forming very different morphologies. They have developed a comprehensive plot of different dealloying systems for both processes of the ligament diameter versus the inverse homologous temperature. The simulation of the microstructural evolution, including techniques such as kinetic Monte-Carlo for ECD and phase-field modeling, is its own field of research, which is beyond the scope of this review. We limit this review to the description of a microstructure, as it results from an experiment or from theory that could represent a snapshot in a series of 3D images that evolve over time. Once we are able to provide a comprehensive set of descriptors for such a microstructure, together with a prediction of its macroscopic properties, we have the tools in our hands for combining the structural and mechanical data toward forming the bigger picture. This can then also be expanded to a dependence of time—whenever this is of interest. 

As input for the structure–property relationship, the morphology can be assessed from different perspectives. For example, one very fundamental question is the self-similarity of nanoporous metals during coarsening. Self-similarity is understood as “whether the morphologies of the feature at consecutive coarsening times are only scaled with the feature size” [33]. Self-similarity is not only relevant for the comparison of microstructures that evolve during coarsening—this is also an important assumption used for the translation of tomography data to micromechanical models, which are then used to predict and compare the macroscopic mechanical response that has been measured for another sample as it was, for example, done in [72]. In this sense, we lay our focus in the following sections more on the question of self-similarity and less on other aspects, such as the active diffusion process (bulk vs. surface), different material systems, and processing routes, as well as the crystallographic orientation during structural evolution.

Various morphological parameters can be derived to quantitatively describe a bicontinuous structure. Measures such as the solid fraction φ and the surface to volume ratio $S_{V}$ represent global parameters. Consequently, such global measures are very robust and are preferred inputs for structure–property relationships. In nanoporous metals, Rösner et al. report a small number of dead ends [16] which influences the load-bearing capacity—i.e., effective solid fractions of the structure (Section 2.4.3). Another global measure of the connectedness of a structure is the genus—i.e., connectivity (Section 2.4.4). We not only discuss these robust scalar parameters as found in the literature, but also shed light on the necessity of precise definitions. 

With the advances in characterization and micromechanical modeling, the available information can be enriched by adding further characteristics toward local feature distributions, such as surface curvatures (Section 2.4.2), ligament diameter (Section 2.4.5), and length distributions (Section 2.4.6). Such parameter distributions are especially important for the creation of artificially generated simulation models. Eventually, non-local information may consider neighboring values such as the determination of the ligament shape and its variation within an ROI (see Section 2.4.7). The challenge with these is the reliability of the measures, as they often strongly depend on the chosen algorithms [73], which we discuss in Section 2.4.5, Section 2.4.6 and Section 2.4.7.

While research went forward, several terms and notations have often been introduced for the same parameters, while in some cases different types of scaling have been used. This can lead to confusion when parameters are compared between two or more works of different authors. As Lilleodden and Voorhees state [5]: “The large spread in reported values for measured elastic modulus of np-Au must point to either the variety in the structure of samples from many groups using a range of dealloying and coarsening procedures, or in the measurements, analyses, and assumptions made in characterizing the structure and structure–property relations.” This statement perfectly summarizes the current challenges we are facing in the nanoporous metals community—they occur in the same way in the development of models, when moving from fitting a single experiment up to powerful tools of increasing the predictive capability along with an increasing number of parameters.

We organized the discussion along the lower column of Figure 1 from left to right, starting from the global measures derived from volumetric or surface information (Section 2.4.1, Section 2.4.2 and Section 2.4.3) and moving successively down to a description of individual features by distributions of local (Section 2.4.5, Section 2.4.6) and non-local measures (Section 2.4.7). For a better coherence of this review, we use a consistent notation of variables that can deviate with the notation in the individual papers. 

#### 2.4.1. Characteristic Feature Size and Length Scale

Most commonly, the structure of nanoporous metals is described as a network of interconnected ligaments and nodes. While in its details the network appears random, global measures of the characteristic feature size as scalar values can be derived in several ways. This forms the foundation for the scaling and hence the comparison of samples of different size, fabrication, or composition. Moreover, this results in the investigation of size effects that relies on self-similarity over a large range of characteristic sizes. 

Fujita et al. have applied the Fourier transform to their TEM 3D reconstruction [26]. From the Fourier transform pattern and the intensity profile, they have extracted a characteristic length scale of L=30 nm. This is associated with a statistical quasiperiodic length of the structure. It resembles the pattern in which the structure is periodically continued—i.e., corresponding to the sum of the average ligament diameter and the average pore diameter $L=\langle d_{lig}\rangle +\langle d_{pore}\rangle$ [26]. This measure is similarly used by Soyarslan et al. and Li et al., who created bicontinuous structures by the Cahn’s method of generating a Gaussian random field using leveled wave functions [47,65]. They have interpreted the characteristic spacing $\tilde{L}$, which is computationally the distance between the maxima in the random field function as the mean distance between local centers of the solid or the pore space. If the solid fraction is φ=0.5, this results in the mean ligament diameter being equal to the mean pore diameter, both $\langle d\rangle =\tilde{L}/2$. For the estimation of the local ligament and the local pore diameter, see Section 2.4.5.

Commonly used and simple to extract is the robust scalar parameter of the inverse specific surface area—i.e., the characteristic length, mostly denoted as $S_{V}^{-1}$ [17,18,33,34,74,75]. It is often associated with the mean value of the distribution of the local ligament diameter 〈d〉, see Section 2.4.5. However, it is often unclear which volume was used for calculation. In a porous structure, the volume can refer to the total foam volume or just the volume of one phase, typically the solid metal phase. Different definitions exist, which are often imprecise:

- surface area per **volume**, $S_{V}$ [17]
- specific area calculated as the ratio of solid (gold) surface to **solid volume**, $S_{A}$ [33]
- inverse of the interfacial area per **unit volume**, $1/S_{V}$ [74]
- reciprocal of interfacial area per **volume**, $1/S_{V}$ [75]
- specific surface area, $S_{V}$, defined as the surface area per **unit volume of the phase** (solidAu) [34]
- surface area per **unit volume of gold phase** used as the characteristic length scale of the structure, $S_{V}$, [18], p. 120
- surface area per **unit foam volume** used for scaling the genus density, $S_{Vf}$ [18], p. 121

As there are obvious reasons for using one or the other definition, it is important to clearly state by which volume the surface area is normalized. If desired, the translation to the surface area per solid volume $S_{Vs}$ can be easily obtained using $S_{Vs}=S_{V}/\varphi$, where $S_{V}$ is the surface area per volume (including solid and pore). Having a common definition for $S_{V}$ is of particular importance, when $S_{V}^{-1}$ is used for the computation of the scaled genus density, see Section 2.4.4. 

#### 2.4.2. Interfacial Surface Curvatures

**Figure 5:**
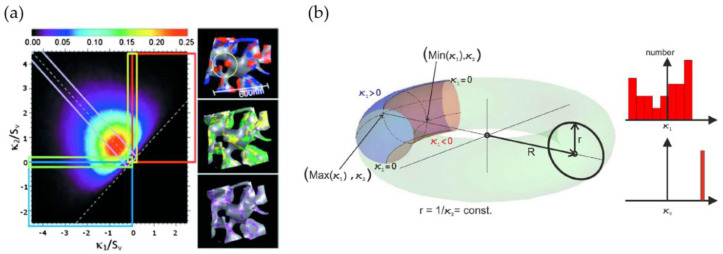
(**a**) Interfacial shape distribution (ISD) plot with near zero average mean curvature, and highlighted surface patches of convex (red) and concave (blue) regions, transition regions (yellow, green), zero mean curvature regions (purple) by Chen et al. [17]; (**b**) schematic ring with inscribed curvature parameters $\kappa_{1}$ and $\kappa_{2}$ by Ziehmer et al. [42]. Reprinted from [17] with the permission of AIP Publishing and [42] with permission from Elsevier.

Meshing of the 3D-reconstructed sample volume allows for an analysis of the distribution of curvatures and the surface normal by utilizing individual surface patches. Commonly, the results are presented as the interfacial shape distribution (ISD) and interfacial normal distribution (IND). The ISD gives the probability to find an interfacial surface patch with a pair of principle curvatures $\kappa_{1}$ and $\kappa_{2}$. In Figure 5a [17], the highlighted surface patches in the inserts schematically show regions with convex and concave shapes (red, blue), transition regions (yellow, green) and zero mean curvature regions (purple). For more details on the ISD quadrants, the reader is referred to the Figure 7 in [76]. Common variables are the principle curvatures $\kappa_{1}$, $\kappa_{2}$, the Gaussian curvature K, and the mean curvature H. In their review, Lilleodden and Voorhees summarized the common variables and equations for curvature calculation and the key structural aspects of curvature [5]. 

Concerning the interfacial curvature, spinodal-decomposed structures show an average mean of zero. Besides the average, the distribution of convex, concave, and saddle-like features of the nanoporus structures can be analyzed. For example, Rösner et al. have found, on average, a convex surface for a sample with a solid fraction of φ=0.24 [16]. Fujita et al. and Chen et al. observed a near zero average mean curvature surface for np-Au samples, see Figure 5a, with each having the same solid fraction of φ=0.5 [17,26]. Chen-Wiegart et al. have observed non-symmetrical ISD plots around the zero mean curvature line for φ=0.28, where mostly saddle-shaped and convex surfaces were present in the structures [33]. The curvature analysis can provide additional insights into the evolution of a microstructure. For instance, local positive Gaussian curvatures resemble pinch-off events [17]. Rösner et al. highlight that surface normal evaluation can show possible anisotropy of the structure, which can be related to the fabrication and corrosion direction [16].

In their review, Lilleodden and Voorhees have revised the question of self-similarity of nanoporous structures, deeply focusing on works analyzing curvature ISDs, besides ligament size and scaled connectivity density [5]. They have collected several works stating evidence for self-similarity and conversely disproving self-similarity. To make meaningful statements about self-similarity during coarsening, it must be ensured that the samples satisfy the conditions of a representative volume element (RVE), which might be lacking in earlier works [5]. 

Besides the structural evolution analyzing average values, the description of the non-local shape is another research topic. Ziehmer et al. have differentiated the nanoporous structure with respect to the ligaments themselves, and inspired by the shape, higher-level structural elements in the form of torus-like rings formed by the interconnected ligaments [42]. They propose to relate mean ligament and ring diameter each to one of the two inverse mean principle curvatures $\langle \kappa_{2}{}^{-1}\rangle$ and $\langle \kappa_{1}{}^{-1}\rangle$, respectively, as these two measures are in correspondence with the shape of a torus surface, as shown in Figure 5b. For the torus, $\kappa_{2}$ reflects the constant inverse ligament radius 1/r, whereas the ring size R is assumed to be reflected by the principle curvature $\kappa_{1}$. Ziehmer et al. found linear dependencies between the mean ligament diameter $\langle D_{lig}\rangle$ and the ring diameter $R\left(\kappa_{1}\right)$ as well as the mean ligament diameter $\langle D_{lig}\rangle$ and the ligament diameter $r\left(\kappa_{2}\right)$. Thereby, they concluded that one of the parameters is sufficient to characterize the structural size. 

As another material system, nanoporous stainless steel was analyzed by Zhao et al. through liquid metal dealloying (LMD) using X-ray nano-tomography [77]. The authors report that the ISD plots are qualitatively similar to the ISDs of np-Au. Further, they observe that samples with increasing solid fractions show a morphological transition from a classic bicontinuous structure to a channel-like porous structure. 

The ISD is frequently used in the analysis of sample structures, whereas it has found no application in the analysis of artificially generated structures yet. As it provides important integral information on the surface shape distribution, it could be a valuable descriptor for the design and validation of artificial structures that are targeted to approach experimental structures as closely as possible. While the question of the comparability of different structures is an unsolved problem, to our best knowledge no one has tested the ISD for such an application.

#### 2.4.3. Effective Load-Bearing Structure

The investigation of nanoporous structures usually assumes these as a strut-like ligament connection between two nodes. However, Rösner et al. have already reported a small number of dead ends, which are only one-side-connected ligaments [16]. By their definition, these are at least as long as they are wide, and it is cautiously stated that shorter dead ends might be seen as skeletonization errors. Later, Hu et al. confirm the existence of dangling ligaments in the FIB-SEM tomography of coarsened np-Au samples [19]. After the skeletonization of the structure, they have removed any singly connected ligaments that are not part of a closed ring. This pruning does not change the connectivity, as no rings are opened by removing the dangling ligaments. Therefore, no essential connections are cut, while excess bulbs are smoothed. For an estimation of the remaining mass active in load-bearing rings, they approximate the volume contribution of each of the straight connections in the skeletonized network. The modified ligaments are simplified as cylinders with an average diameter 〈d〉 and average length 〈l〉. Depending on the assumed nodal overlap, they find the solid fraction of this ring network to be about 10–14% instead of 30% solid fraction of the initial structure. This means that not even half of the initial structural solid volume contributes to load bearing. 

**Figure 6:**
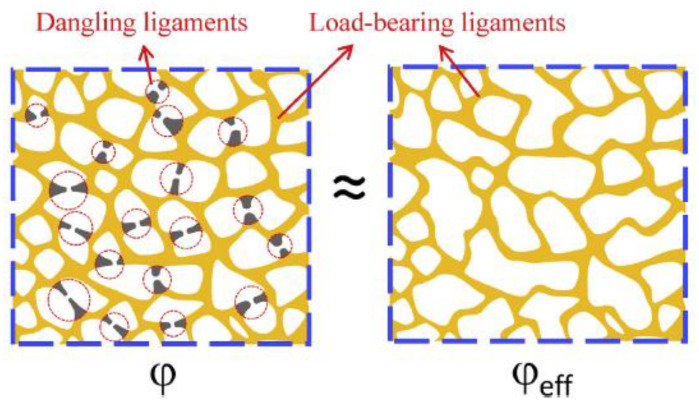
Schematic of removing dangling ligaments from the initial structure, results in structure with all load-bearing ligaments at the effective solid fraction $\varphi_{eff}$, by [78]. Reprinted from [78] with permission from Elsevier.

Similar results are reported by Liu et al., who have inversely determined the effective solid fraction $\varphi_{eff}$ from macroscopic compression testing by inserting the measured Young’s modulus in Equation (1). Their results suggest $\varphi_{eff}=0.25\varphi$ for the tested sample—i.e., only 1/4 of the mass contributes to the load transfer [78]. Further, the ratio $\varphi_{eff}/\varphi$ depends on the solid fraction, ligament size, and applied plastic strain of the sample. The schematic drawing in Figure 6 shows the presence of dangling ligaments at a solid fraction of the initial structure φ, and the reduced structure with actually load-bearing ligaments at an effective solid fraction $\varphi_{eff}$ [78]. Along with observations in the literature, Mameka et al. have developed the concept of load-bearing rings that can explain the discrepancy between the measured and predicted mechanical properties using the Gibson–Ashby scaling laws [79]. For more details on the implications of the effective load-bearing structure on the macroscopic mechanical properties, see Section 3.3.1.

At the boundaries of the RVE, the determination of dangling ligaments and their treatment for the calculation of the genus are particularly difficult for an RVE of limited size [19], see following Section 2.4.4. Richert und Huber differentiated between boundary dangling, dangling, and fully interconnected ligaments. For the boundary dangling ligaments, it is unclear whether they should be counted as interconnected or dangling fraction [50]. The importance of these ligaments becomes evident when comparing the rate of convergence of macroscopic mechanical properties with that of the scaled genus density. For achieving convergence of the scaled genus density, an RVE is required, which is about 10 times larger in edge length, than the RVE needed for achieving convergence of the macroscopic mechanical properties [80]. 

#### 2.4.4. Connectivity and Genus

The topological descriptors can be divided into global and local measures. As defined in basic graph theory, the local measures are denoted as nodal degree, valence, or coordination number which can be extracted from the skeleton. For more details on the elements in algebraic topology, see [81]. The most common global measure is connectivity, which is the “number of closed-loop cuts that can be made on a surface, while leaving it connected as one piece.” In theory, genus and connectivity can differ when the solid contains internal pores. To simplify the topology parameters, Hu et al. assume possible internal pores in their np-Au samples to be zero—this leads to genus and connectivity becoming equivalent terms [19]. In this sense, the terms genus and connectivity are used equivalently in this review. The genus is also interlinked with the Euler characteristic [82] and the Betti numbers [81]. For a more detailed discussion, see [19]. 

Connectivity can either be evaluated using a triangulated surface mesh or from the skeleton made up by interconnected nodes and vertices. Hu et al. highlight a connectivity evaluation at the finite volumes boundary because any possibly extending connections are cut there. The correct value of a structure’s connectivity falls between the following two extrema: (i) connecting all nodes that intersect the surfaces and (ii) assuming the finite volume is free-standing. With increasing volume, these two extrema converge to each other [19]. An algorithm developed by Odgaard and Gundersen is implemented in the Fiji plugin BoneJ and accounts for this so-called “edge effect” [83]. 

Mangipudi et al. have analyzed the FIB-SEM tomographic reconstructions of np-Au and, for comparison, a model generated spinodal structure (phase-field Cahn-Hillard) and gyroid structure, all with the same solid fraction φ=0.32 as the np-Au samples [34]. As the results in the simulations of stress–strain curves differed unexpectedly, the authors conducted morphological and topological characterizations. For the analysis of local topology measures, they have counted the connectivity—the number of ligaments that are connected to each node of the skeleton—and found a similar nodal connectivity for all models. The majority of the nodes were three-fold connected, while the minority were four-fold connected, which could not explain the mechanical differences. In contrast, the scaled genus density $\bar{g}$ as another topological parameter characterizing the overall connectivity showed very different values for the three structures. For an in-depth discussion on the connectivity and its influence on the mechanics resulting in new scaling laws, see Section 3.3.

Following our discussion in Section 2.4.1, about a precise definition of the used volume to calculate the inverse specific surface area $S_{V}^{-1}$, it is of interest to discuss the possible normalizations for the scaled genus density. Chen et al. have integrated the Gaussian curvature for the genus calculation [17] using the Gauss–Bonnet theorem [84]. The sample with φ=0.5 showed a genus per unit volume of $g_{v}=48\;\mu m^{-3}$. For providing a dimenionless genus, $g_{v}$ is scaled by ${\left(S_{V}^{-1}\right)}^{3},$ yielding $g_{v}S_{V}^{-3}=0.147$. Chen et al. have stated that this value is far off the scaled genus of a minimal Schoen’s G surface, but close to the value 0.13 of a simulated bicontinuous structure using spinodal decomposition, which is constant during coarsening [74].

**Figure 7:**
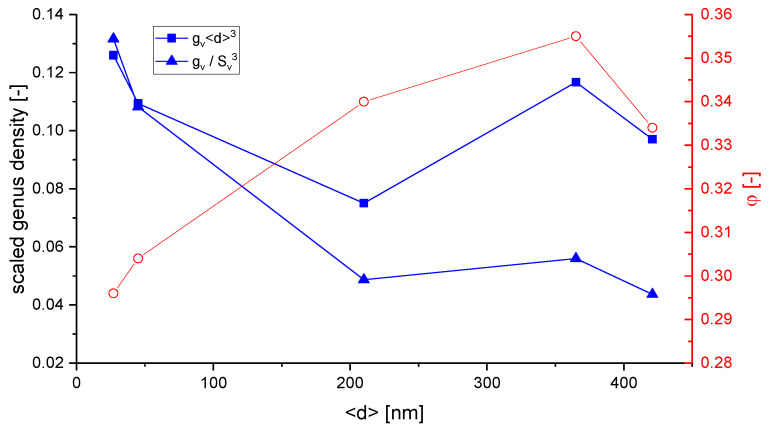
Comparison of scaled genus density from data of [36] as $g_{v}{\langle d\rangle }^{3}$ as scaled in [19] and for the scaling $g_{v}S_{V}^{-3}$ as used in other works [17,75].

Hu et al. have suggested a normalized $g_{v}$ in the form $g_{v}{\langle d\rangle }^{3}$, which yields a value of around 0.1 for samples with mean ligament diameters from 〈d〉=26 to 420 nm [19]. The scaling of the data [36], Table 4.2, according to $g_{v}S_{V}^{-3}$ is shown together with the $g_{v}{\langle d\rangle }^{3}$ data in Figure 7. Although the characteristic lengths 〈d〉 and $S_{V}^{-1}$ show a linear dependence, the scaling with a power of three causes a high level of sensitivity to the effect of volume shrinkage which is visible in the rising value of φ. This seems to affect $S_{V}^{-1}$, and in contrast to $g_{v}{\langle d\rangle }^{3}$, the scaling $g_{v}S_{V}^{-3}$ would not allow for a conclusion of self-similarity as it was drawn based on $g_{v}{\langle d\rangle }^{3}$. The fundamental question arises as to which way of normalization allows for a topological comparison of structures that are not necessarily self-similar. 

Besides the issue of genus scaling, an important finding by Liu et al. shall be highlighted here regarding self-similarity [78]. In comparison with Hu et al., who observed a constant scaled connectivity density and geometric self-similarity during the coarsening of their np-Au samples with a solid fraction that varies from φ=0.30 to φ=0.34 [19,36], Liu et al. could provide evidence for a reduction of the network connectivity during the coarsening process at φ=0.26 [78]. For this solid fraction, they could show that the macroscopic Young’s modulus decreases significantly with coarsening time. In their following work, Liu and Jin have analyzed samples with different initial solid fractions of φ=0.46, φ=0.35 and φ=0.26 [85]. All samples were coarsened from initially 4 nm up to 600 nm in ligament diameter, see also [6]. For the sample with a solid fraction of φ=0.35, the observed loss is less significant—no change of macroscopic Young’s modulus could be observed at φ=0.46. For self-similar coarsening, constant elastic properties are expected. Therefore, this study shows that self-similar coarsening is present only for the higher solid fractions and that previous statements regarding self-similarity are limited to the specific solid fraction of the sample under investigation. 

**Figure 8:**
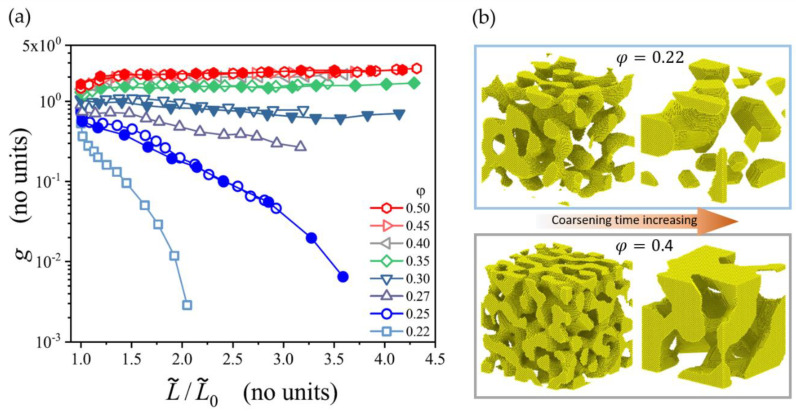
(**a**) Scaled genus plotted over the characteristic length of nanoporous structures calculated by kinetic Monte Carlo (KMC) simulations. Self-similar coarsening for the solid fraction φ>0.3 is observed [47]. (**b**) Structure with φ=0.22 losing connectivity by breaking up into clusters, whereas the structure of φ=0.4 coarsens self-similar with increasing time [47]. Reprinted from [47] and available under the terms of the Creative Commons Attribution 4.0 International license (CC BY 4.0).

Recently, Li et al. have computationally studied the coarsening process of microstructures generated by leveled Gaussian random fields by using the kinetic Monte Carlo simulation [47]. In their extensive study, the solid fraction of the microstructures ranges from φ=0.22 up to 0.5, which represents the full range of relevant solid fractions. The network connectivity is measured by a scaled genus density as $g=G{\tilde{L}}^{3}/\;V_{total}$. Confirming the results by Liu and Jin [85], the scaled genus density stays invariant during the coarsening of samples with φ≥0.3, but it decreases for samples with a lower initial solid fraction φ<0.3. This is illustrated in Figure 8. At the lowest solid fraction 0.22, the structure breaks up into clusters and the scaled genus density approaches zero. For the structure with the solid fraction of 0.4, the coarsened structure appears like a zoom-in at the initial structure and visually confirms self-similarity. 

It is, therefore, evident from experimental and modeling results that self-similarity during coarsening can be only expected for solid fractions φ≳0.3−0.33. Consequently, a comparison between the results of different groups is rather simple in this higher range, while for φ<0.3 everything depends on the coarsening, which significantly complicates the situation. This is also important for other morphological parameters, such as the interfacial surface curvature, or the diameter distribution. For an attempt to bring together mechanical data from different sources over a large range of solid fractions, see Section 3.3.3.

#### 2.4.5. Ligament Diameter Distribution

In 1992 Li and Sieradzki presented normalized histograms of the void-size and the ligament-size based on 2D SEM image analysis. A small increase in the distribution width was found toward larger ligament sizes—i.e., higher annealing temperatures [25]. Rösner et al. have performed a stereological analysis based on granulometry, resulting in the volume-weighted local feature size [16]. They highlight the broad distribution of ligament shapes and diameters that range from 5 to 30 nm, with an average of 16 nm for the solid phase and 28 nm for the pore phase. They show an agreement of the solid fraction φ=0.24 and the simple structural equation for porous materials φ≈ ${\left(\mathrm{ligament}\;\mathrm{radius}/\mathrm{pore}\;\mathrm{radius}\right)}^{2}=0.32$. Fujita et al. have determined the diameters of gold ligaments or pores by counting the removed pixels from the surface to the skeleton center-line (surface thinning), where the diameter distributions were found to follow a more Gaussian-like distribution with an average value of 〈d〉=11 nm [26]. 

The Gaussian distribution is the most common distribution used to evaluate diameter histograms, next to Weibull fits and other measures such as statistical T-tests. The diameter distribution is mostly used to study self-similar evolution during coarsening—many studies have confirmed this. Hu et al. have applied the Biggest-Sphere thickness algorithm to their 3D reconstructed RVE to identify the mean ligament diameter and the scaled diameter distribution at different coarsening stages [19]. Self-similar coarsening is indicated by a very good fit of the Gaussian distribution that shows nearly no broadening or shift of the curves. Tse et al. have investigated the possible differences of ordered and disordered np-Au during dealloying [86]. Instead of fitting a Gaussian distribution, they have used statistical T-tests on ligament diameter distributions. They have found no significant deviation in the distributions, which suggests no substantial difference between the surface diffusion of gold on ordered or disordered surfaces. Stuckner et al. have analyzed the influence of annealing on the ligament diameter using Weibull vs. Gaussian fits, and no significant difference has been found [87]. 

The main concern when comparing the results between different groups with regard to the ligament diameter is the question about where and how it was estimated. McCue et al. have used their developed tool AQUAMI to data mine 2D np-Au images of various publications [45]. They have pointed out the difficulty and resulting systematic discrepancies while comparing the results gained by different measuring approaches—ranging from manual measurements to automated computational estimations. Even in manual assessment, the variation ranges from measuring the smallest parts of the ligaments to measuring the statistical cross-sections.

From computational image analysis, one might expect results with well-defined thickness values. It is, however, very important to understand how different algorithms work, especially when analyzing complex nanoporous structures. As discussed in Section 2.3.3, Richert et al. have found for nanoporous gold that the biggest-sphere Thickness algorithm systematically overpredicts the local thickness assigned to a certain skeleton voxel. It is shown that the biggest-sphere Thickness algorithm tends to smear local thinning by propagating the ligament thickness from thicker into the thinner parts [50,53]. Furthermore, Richert et al. have shown, with the help of artificially created model structures, that the Euclidean distance transform (EDT) systematically underestimates the local thickness. For example, the same tomography data from [19] yields different average thickness values of approximately 400 nm (Th) and 300 nm (EDT) [53], see Figure 4c. 

In their study, Mangipudi et al. provide an overview of four different measures of the ligament diameter for samples produced by electrochemical corrosion, free corrosion, and annealing with φ≈0.3 and ligament sizes ranging from d=30–85 nm [88]. In addition to the mean intercept length d measured in SEM images and the inverse specific surface area $1/S_{v}$, they have used a directional pair correlation function (PCF). Evaluated in different directions, the latter can reveal structural anisotropy. All the values give different values for the same structure—it is expected as they have different fundamentals, but they show consistent trends among the four analyzed samples. Thus, the authors conclude that the four diameter measures are simply related by constants. 

A similar investigation is provided by Jeon et al., where in addition to the average ligament size d measured from SEM images, the inverse specific surface area $1/S_{v}$, and the volume-weighted biggest sphere thickness 〈D〉 are determined. The solid fraction is also φ≈0.3, and extends the range investigated in [88] from d=70 nm to 710 nm [43]. Both works independently found indications that the np-Au coarsened in a self-similar way, as all characteristic length shows mostly linear dependencies that are simply connected by a constant. Further, Jeon et al. could show that normalizing the scaled connectivity density in the form $C_{V}{\langle D\rangle }^{3}$ produces a constant value, which holds over the whole coarsening process [43]. This is in qualitative agreement with the results by Hu et al. [19], but the value of about $8\times 10^{-4}$ is two orders of magnitude lower. The big difference in the results suggests that the samples under investigation are very different in their scaled connectivity density, which tends to contradict the insights into self-similar coarsening at solid fractions of φ≳0.3 that would imply a similar value for the scaled connectivity density. We should, however, keep in mind that φ≈0.3 is just at the border of self-similarity (compare [47,85]), and it is possible that the connectivity can be dramatically reduced if enough time is given. Therefore, the big difference can be caused by a different sample preparation. In addition, this might also be influenced by different ways of measuring the connectivity and diameters. Here modeling could be used to support the quality assurance of the methodology and to gain further insights regarding the interpretation of such data (see Section 3). 

**Figure 9:**
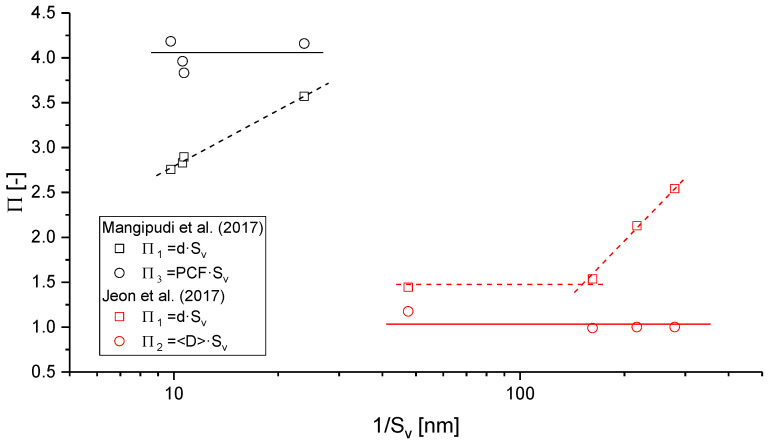
Dimensionless parameters derived from various characteristic lengths provided in the literature and their reliability in the assessment of self-similarity. Data has been taken from [43,88].

To get an impression about various geometric lengths as candidates for a reliable assessment of self-similarity, we can define several dimensionless parameters $\Pi_{i}$. It is preferable to use the inverse specific surface area $1/S_{v}$ as the common basis for the normalization, as this is a very robust measure composed of two global descriptors. The data obtained for the dimensionless parameters $\Pi_{1}=d\times S_{v}$, $\Pi_{2}=\langle D\rangle \times S_{v}$, and $\Pi_{3}=PCF\times S_{v}$ is presented in Figure 9. 

If self-similarity is assumed, all dimensionless geometric lengths should be constant and linearly dependent on each other during the coarsening of a sample, i.e., $\Pi_{i}\propto \Pi_{j}$. While the normalized characteristic lengths $\langle D\rangle \cdot S_{v}$, and $PCF\cdot S_{v}$ support the hypothesis of self-similar coarsening—the results from both papers discussed here show consistently that the ligament diameter d, as determined from the 2D SEM images, behave differently. The inherent uncertainty of this measure reflects in its description [43]: “The average ligament size [d] is the average diameter of necks in connecting ligaments, possibly the thinnest part measured by 2D SEM images.” Consequently, the average local thickness 〈D〉, extracted from the diameter of largest spheres, includes all cross-sections along the ligaments and nodes; it should, according to our understanding, be larger than the ligament size d [50,53]. It is unclear how Jeon et al. could determine values for d that are more than a factor of 2 larger than 〈D〉 [43].

Independent of this detail, we can see from Figure 9 that it is advisable to use the much more robust measures for quantitative analysis, such as $S_{V}^{-1}$, 〈D〉, or PCF. Concerning the local ligament thickness d, it is possible that its size dependency results from uncertainties in the manual approach of image processing or from a change of the ligament shape. In the latter case, the increasing value suggests a trend from highly concave towards cylindrical and even convex shapes. This would imply that the condition of self-similarity does not hold in the strict sense. For a better understanding, a methodology for a systematic analysis of ligament shapes is highly desirable, see Section 2.4.7. 

#### 2.4.6. Ligament Length Distribution

Before we move forward to the description of the ligament shape, we need to introduce the ligament length l, which, in combination with the ligament diameter d or ligament radius r=d/2, defines the aspect ratio of a ligament. This is an important parameter that connects the ligament geometry with the solid fraction for a given structure [9,20,89].

In comparison with the ligament diameter, the ligament length is a parameter that is rather straightforward to classify. Rösner et al. have skeletonized the pore phase and solid phase to estimate the characteristic lengths as the mean skeleton branch lengths of the respective phases, which are $l_{p}=55$ and $l_{s}=17$ nm, respectively, yielding and aspect ratio for the ligaments of $l_{s}/d_{s}\approx 1$ (values at Gaussian peak positions) [16]. Fujita et al. have calculated also the skeleton via surface thinning and measured the ligament and pore canal length, which has shown asymmetric distributions at an average ligament length of 〈l〉=27 nm, for both phases [26]. In contrast to [16], the ligaments and pore canals seem to be geometrically identical, which is due to the solid fraction of φ=0.5 [26]. 

Commonly, nanoporous metals have a ligament length distribution that is asymmetric [26,50,90]. In comparison with a Gaussian profile that gives a good fit for ligament diameter distributions, the mean ligament length is shifted to the smaller lengths and the distribution has a long tail at larger lengths. Therefore, Stuckner et al. have used Weibull and log-normal fits, where the later shows the better fit, with the average shape parameter close to the average mean ligament length [87]. 

The uncertainty about comparing the ligament length between publications is the question of what is defined as “the ligament”. Is the nodal area subtracted or remains included? Is the Euclidean distance measured or the path along the curved ligament axis? For example, Pia and Delogu have measured the ligament length after subtracting the nodal volume extension [91]. Using the skeletonization approach as most research papers do [16,26,50,90], the ligament length is extracted from the skeleton interconnected at 0-dimensional nodal points. Thus, no nodal volume extension is subtracted from the length. Therefore, care needs to be taken if this parameter is used for comparing papers or as a descriptor for modeling purposes.

#### 2.4.7. Ligament Shape Distribution

Beyond the question of self-similar coarsening, the ligament shape is an important descriptor for the creation of artificial structures, which are representatives for nanoporous metals. The aspect ratio characterizes a structure that, on average, is assumed to consist of cylindrical ligaments. It allows for a comparison of different structures and relates to the solid fraction. If we consistently use the radius to length ratio, the literature report r/l values of 0.5 [16], 0.2 [26], or, after subtraction of the nodal extension, 0.66 [91]. According to Roschning and Huber, the model structures with cylindrical ligaments organized in a randomized diamond lattice require r/l=0.27 for a solid fraction of φ=0.3 [92]. This is well within the experimentally observed range. 

Besides the aspect ratio, the variation of the radius along the axis is crucial for predicting the mechanical behavior. Hu et al. have stated next to their diameter distribution analysis: “In the context of the weakest link statistics, the mean ligament size may not be the critical one. Rather, the smallest size is likely to trigger initial failure governing strength” [19]. This is a critical point and its consideration requires the development of techniques that would allow the transfer of real or sufficiently realistic diameter information to a finite element method (FEM) simulation model. However, as only limited tomography datasets are available, this requires an abstraction of the ligament shape with a simple function that can be parameterized and statistically distributed within the range of experimental shapes. 

**Figure 10:**
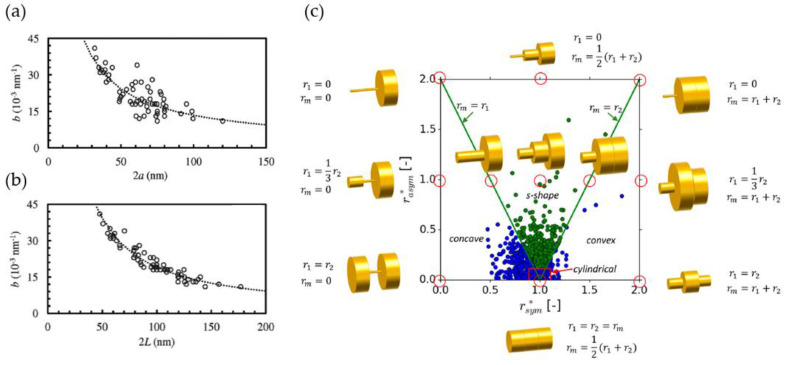
(**a**,**b**) Distribution of concaveness shape factor b for the description of symmetric parabolic ligament shapes from 2D SEM images according to Pia and Delogu [91]; (**c**) the mapping of more general spherical-parabolic ligament shapes from 3D tomography data and description by symmetry parameter $r_{sym}^{*}$ and asymmetry parameter $r_{asym}^{*}$ by Richert and Huber [50]. Reprinted from [91] with permission from Elsevier, and from [50] distributed under the Creative Commons Attribution License (CC BY 4.0).

Concerning the parameterization of the contour of the ligaments, several papers state that a minimum diameter can be observed roughly in the middle, with the increasing diameter approaching the nodes. Pia and Delogu have analyzed 2D SEM images by fitting a parabolic function of the form $r=a+bx^{2}$ to the ligament shape visible in 2D images [91]. The shape factor b provides information about the concaveness (b>0) or convexness (b<0) of the ligaments. The authors, however, just consider concave ligaments and using this fit function, the shape of the ligaments is assumed to be symmetrical, see Figure 10a. They show a linear correlation between 2a (mid thickness) and the ligament length. Further, the measured concavity degree b decreases as the mid-thickness and the ligament length increase. Longer ligaments show less extreme concave shapes—i.e., they are closer to cylinders. The smaller the ligaments, the more concave the shape profile (which means it is closer to pinch off). Essentially, Pia and Delogu conclude that a quadratic function represents a good description of the variation of ligament thicknesses along the ligament length. The broad distribution of the ligament length and the thickness indicates a significant variety of shapes. Besides that, the nodes are quite massive, which supports using the concave parabolic fit function with b>0 in combination with a nodal mass at both ends, which are not part of the ligament, similarly to the unit cell proposed by Liu and Antoniou [93]. 

Inspired by the work of Pia and Delogu [91], Richert and Huber have developed the symmetry parameter $r_{sym}^{*}$, to describe the convexity/concavity of a ligament and also introduced an asymmetry ratio factor $r_{asym}^{*}$ that represents the difference in the node radii at both ends normalized with their average [50]. The two parameters $r_{sym}^{*}$ and $r_{asym}^{*}$ represent two indepent descriptors for the shape of a ligament and allow the mapping of all ligament shapes in a structure in a single plot, as shown in Figure 10c. Analyzing the 3D FIB-SEM np-Au reconstruction provided by Hu et al. [19], they have found a surprisingly high number of asymmetric s-shaped contours: approximately 50% of ligaments are s-shaped and besides 25% are concave, 5% convex, and 25% cylindrical ligaments, assuming that deviations below the 10% ligament radius are accepted as cylindrical. Since almost half of all analyzed ligaments in the np-Au sample are found to have an asymmetric contour, any parameterization assuming symmetrical ligament geometries probably leads to biased statistics in favor of a cylindrical shape [50].

### 2.5. Concluding Remarks on Structural Characterization

In Section 2 we have discussed the parameters used in the characterization of nanoporous metals. In addition to the solid fraction as the most relevant dimensionless parameter, others are geometric lengths or inverse lengths that are determined from global measures as well as local features of the structure. 

In view of the discussions about the ligament diameter and ligament shape, an open and almost untouched field is the facet formation during coarsening, which is visible in experiments (see [94], Figure 9; [89], Figure 2) and predicted via large-scale kinetic Monte Carlo simulations in [95]. As faceting tends to flatten the surfaces of the ligaments along the close packed planes of the crystal, it can be expected that this has an effect on the evolution of the ligament shape during coarsening. One can, therefore, speculate that the probability of cylindrical ligament shapes increases with coarsening time while the number of convex and concave ligaments is reduced.

Among other aspects, self-similarity relates macroscopic mechanical properties to the ligament size and avoids uncertainties in the interpretation of data that may result from the potential differences in the morphology of the sample as it evolves during coarsening. With the improving resolution in nanotomography, advances in image processing, and progress in the translation into computational models that has been made during the last few years, we can accompany the experiment with numerical simulations. This allows for an interpretation of experiments in situations where self-similarity does not apply and opens up the possibility to bring together datasets from different samples and sources. 

Compared to the large variety on characteristic lengths, data on topological measures is scarce. This is due to the still very limited number of existing tomography datasets of nanoporous samples. However, the contributions by Jin and co-workers [78,85] clearly show the important role of connectivity on macroscopic mechanical properties. So far, only a few works compare morphological descriptors for different samples or even a mix of samples and artificial structures. Mangipudi et al. have investigated two np-Au samples, a gyroid, and a spinodal structure of the same solid fraction with respect to characteristic lengths, surface curvature, topology, and mechanical properties [18]. It turned out that the scaled genus density is the most important as it shows a strong correlation with the leading constant in the Gibson–Ashby scaling law (1), which deserves further research in terms of a universal structure–properties relationship, see Section 3.3.3. Therefore, it can be concluded that the pair of parameters (φ, $g_{v}l_{c}^{3})$ represents the dominant input for a structure–properties relationship, where $l_{c}$ is a characteristic length that scales the genus density $g_{v}$ to a dimensionless quantity and should be ideally chosen in such a way that the topology descriptor becomes independent of the assumption of self-similarity. 

Following the literature presented in Section 2, an overview of micromechanical modeling work in this field is given in Section 3. The geometrical descriptors and the macroscopic mechanical response represent the input and output of the equation, thereby forming a structure–properties relationship, respectively. In the investigation of this relationship, we rely on data. Section 2 plainly shows that we are confronted with a large variety of geometrical parameters. Experiments provide valuable, but limited, insights into the underlying dependencies—particularly then numerical simulations are a powerful tool for pushing the limits. Section 3 gives an overview of the literature in micromechanical modeling of nanoporous metals. It impressively reveals how much this field benefits from the knowledge gained by experimental work. Further, emphasis is placed on how this method helps to widen our limited view and how questions brought up in the experiment can be answered.

## 3. Micromechanical Modeling

**Figure 11:**
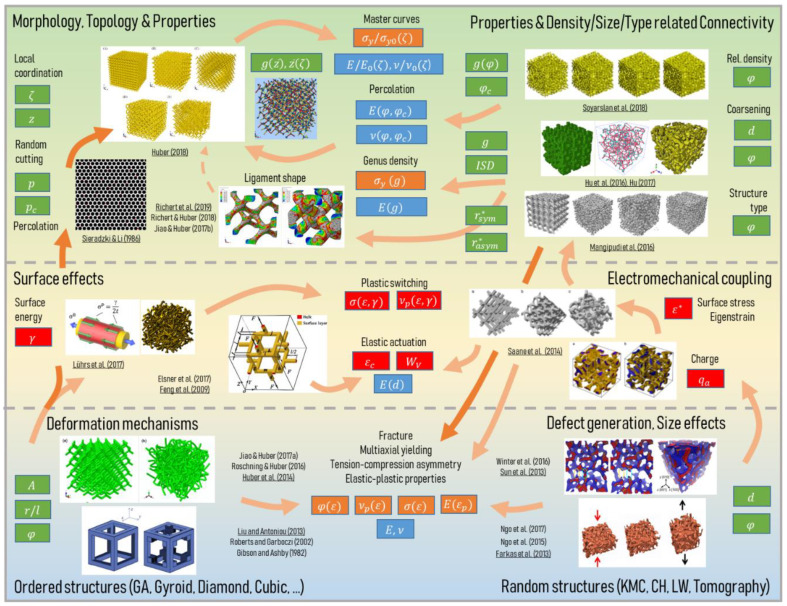
Overview of micromechanical modeling of nanoporous metals. Sources of the images are underlined. For figure reprint permissions, see Note at the end of the paper.

In many aspects, the micromechanical modeling of nanoporous metals is very similar to the modeling of open pore foams where the methodology is mainly based on FE solid models and FE beam models [20,22,23,24,96]. Elasto-plasticity and deformation localization is investigated using various unit cells, including Gibson–Ashby, simple cubic, reinforced body-centered cubic, body-centered cubic, Kelvin, diamond, and Weaire Phelan [97,98]. Excess volume from the overlap of beam elements connecting in nodes was removed in FE beam models by a modification of the ligament shape [22]. This is sufficient for the correction of the nodal mass for solid fractions of up to 10%. The effect of structural irregularities is studied with random Voronoi foam models, periodic cells with randomly deleted features, or perturbation of the structure [20,96,97,99,100]. An advantage of foams is that they show structural feature sizes in the order of 100 µm, thereby providing better access to all the relevant geometric details via SEM and micro-CT for which ligaments and nodes could be modeled in all geometric details [22,24]. Therefore, compared to nanoporous metals, the characteristics of the ligaments and nodes were incorporated in foam modeling much earlier and there still exists significant knowledge in the foam community that would be worth investigating also for nanoporous metals. For instance, Harb et al. have investigated the energy distributions in aluminum and polymeric foams modeled with different cell structures; they have nicely shown that, in addition to axial bending as the dominant deformation mechanism, other modes play a role, such as shear-torsion or axial torsion [101]. They have found that the contribution of torsion increases with increasing perturbation of the structure. About four years later, the usually unnoticed role of torsion as a relevant deformation mechanism in nanoporous metals was reported in [102,103]. It is fascinating to see how research converges to the same result, although the starting points, the ideas behind, and the methods for approaching the problem can be very different. In this sense, we will see that the micromechanical modeling of nanoporous gold evolved along two initially disconnected paths, which tend to grow closely together today, as indicated by the left and right column of Figure 11. 

Over more than a decade, the interpretation of experiments in the nanoporous metals community was based exclusively on the Gibson–Ashby scaling laws, which are based on ligament bending as the main deformation mechanism of open pore materials [7]. Until today, important insights are gained with the help of this model, which is robust and simple to use [14,78]. Micromechanical models originating from Gibson–Ashby and other periodic structures are arranged along the left column of Figure 11 from bottom to top. Computational groups in the nanoporous metals community used also other methods for RVE generation—phase field methods [104], kinetic Monte Carlo simulations [105], or structures generated by Gaussian random fields [20,106,107]. Each delivers structures with striking similarities to nanoporous metals. Developments based on such microstructures are included in the right column of Figure 11, together with micromechanical models based on tomography data.

For the simulation of the elastic–plastic deformation behavior of a nanoporous metal, molecular dynamics (MD) happen to be the natural choice. Here the open-source code LAMMPS [108] with the EAM potential [109] is widely used. This technique offers numerous advantages over finite element methods (FEM). It incorporates surface effects and finite size effects in a natural way via the chosen potential. However, MD is computationally demanding. For achieving solutions in a reasonable time, the size of the simulation box and the ligament size are typically limited to 100 lattice spacing in edge length and d~5 nm, respectively. The challenge arises how to bridge the gap from MD simulation of structures with few nm ligament size and limited cell size to ligaments of about 100 nm that can be found in coarsened specimens [19,79]. Here the obvious questions are: How far can we go up with MD and how far can we go down in the scale with continuum FE? What can we learn with each method and how can the results be validated and compared with the experimental findings? How can we overcome limitations that are naturally inherent in experiments and simulations to gain more detailed insights and, at the same time, a better overview of the effects within a big parameter space? 

In the following sections, we organize the relevant literature dealing with the micromechanical modeling of nanoporous metals along the overview presented in Figure 11. In three sections we address the prediction of the macroscopic elastic–plastic deformation behavior, the integration of the effects of surfaces stress and surface energy, and the discussion of the effects of topology on the macroscopic response of the material. The works on the left column of Figure 11 cover RVEs based on periodic cells, whereas the right column summarizes works that are mainly based on random microstructures. Both paths contribute pieces of a deeper understanding of the structure–properties relationships, as indicated in the middle column. 

As far as scaling laws are concerned, such aspects are only included if new insights are gained with the help of numerical models. Extensions of Gibson–Ashy scaling laws that are motivated from experimental works are not considered here. 

### 3.1. Elastic–Plastic Deformation Behavior

In the following, we will focus on the modeling of nanoporous metals on the nanoscale using molecular dynamics (MD) and finite element methods (FEM). Besides the structure, the elastic–plastic deformation predicted with MD is governed by the chosen potential and the definition of the macroscopic loading. On the macroscopic level, the macroscopic stress–strain curves, the evolution of elastic and plastic Possion’s ratio, as well as the porosity can be obtained. Further, the atomistic arrangement can be analyzed with respect to the emission of dislocations and formation of plane defects, such as stacking faults and twins. This provides insights into the mechanisms that play important roles in terms of defect generation and storage and the role of surface effects for elastic deformation and plastic yielding. As it has been demonstrated for foams, FE simulations provide a continuum perspective with a high variability of structures and material laws. The challenge is to decide on how to model each of them and, therefore, the calibrated material parameters are connected to the chosen geometrical description of the structure. 

First works on elastic–plastic deformation consider tension and compression, whereas later works focus on one direction. Typically, modeling work carried out in close cooperation with experimentalists commonly deals with macroscopic compression. In contrast to experiments, simulations can predict elastic–plastic deformation during tensile loading including ductile fracture. Therefore, the literature summarized in the following is not strictly chronologically sorted, but thematically arranged along the research on combined tension/compression, pure compression, and the pure tension of nanoporous metals. 

#### 3.1.1. Tension/Compression

**Figure 12:**
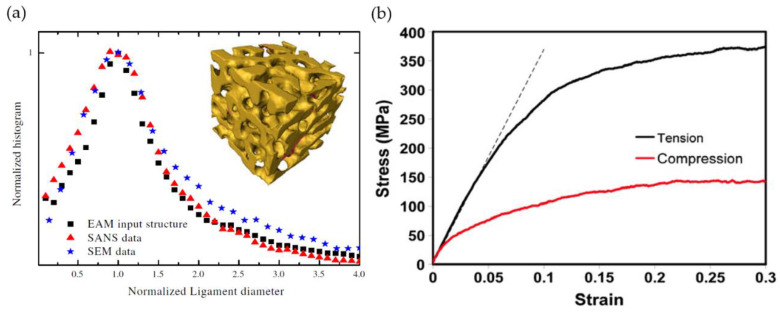
(**a**) Model of np-Au by Crowson et al. [111] and (**b**) the predicted stress–strain curve for tension and compression, showing significant asymmetry due to surface effects by Farkas et al. [112]. Reprinted from [111] and [112] with permission from Elsevier.

The literature on the atomistic simulation of bicontinuous nanoporous metals dates back to [110]. Structures with the solid fractions φ=0.5 and periodic boundary conditions were generated with a phase field model. Applying the Johnson and Trimble potentials for gold, the magnitude of the surface relaxation and its effect on the macroscopic relaxation is determined. It is found that, in addition to the curvature dependent mean pressure given by the generalized capillary equation, the geometric volume change of np structures depends on the surface relaxation. An extrapolation of the simulation results to the estimated surface-to-volume ratios of experimental samples predicts an additional strain of $2\times 10^{-5}$, which is found to be in good agreement with the experimental results of $1\times 10^{-4}$. An 18 nm × 18 nm × 18 nm cube sample of np-Au with average ligament diameters of 1.3, 1.4, 1.5, 1.8, 2.1, 2.7, and 3.6 nm was studied by Crowson et al. [111]. The normalized ligament diameter distribution of the phase field structure showed excellent agreement with experimental distributions obtained by small-angle neutron scattering (SANS) and digitized scanning electron micrographs, see Figure 12a.

Owning to the small ligament sizes at and below the lower edge of experimental values, the effects of surface stress were found to be most significant. Scanning through potentials with increasing surface stress initiated plastic deformation with the formation of stacking faults at the surface stress of 3.2 (100) and 4.5 (111) J/m^−2^, causing a mean pressure of 3.7 GPa in samples with ligament diameters equal or higher than 1.5 nm. The simulations showed that spontaneous plasticity is observed, driven solely by the surface stress and without any external load [111]. 

With this model set-up, Farkas et al. have investigated the elastic–plastic deformation behavior of np-Au foams under external tension and compression loading [112]. From the macroscopic response, Young’s modulus of E= 3.7 GPa and an asymmetry in the yield strength of $\sigma_{y}=$ 370 and 140 MPa were observed under tension and compression, respectively, which is very significant. The deformation went along with significant dislocation activity, forming stacking faults, and nanotwins. Unexpectedly, in tension, a densification of the sample is observed that corresponds to a larger value of the plastic Poisson’s ratio, starting from $\nu_{p}=$ 0.33 and rising up to 0.6. This is accompanied by a decrease in the total pore volume fraction from 75% to 71%, while the average pore size decreased by 12% due to plasticity inside the ligaments and pore collapse. During macroscopic compression, the plastic Poisson’s ratio is predicted close to zero. This is caused by an average tensile stress of 3.2 GPa in the outermost layer of surface atoms that cause a compressive stress of 1.9 GPa inside the ligaments. 

The residual compressive stress in the ligaments finally add to the applied external stress, thereby promoting early plastic yielding under compression and working against plastic yielding in tension, as visible in Figure 12b.

Farkas et al. have applied a Hall–Petch law that predicts significant tension/compression asymmetry up to 20 nm ligaments. For ligament sizes larger than 10–20 nm, the model recovers a symmetric tension/compression behavior. Further, analyzing the macroscopic yield strength with Gibson–Ashby scaling law, a reasonable agreement for the elastic modulus is found. Concerning the yield strength, the result under tension implies that the yield strength of the ligaments should be 4.3 GPa, which corresponds to results from Au nanowires under tension and approaches the theoretical yield strength. Lacking corresponding experimental studies, Farkas et al. have pointed out that novel experimental methods might allow the test of the existence of tension/compression asymmetry in nanofoams in the near future [112]. 

#### 3.1.2. Macroscopic Compression

**Figure 13:**
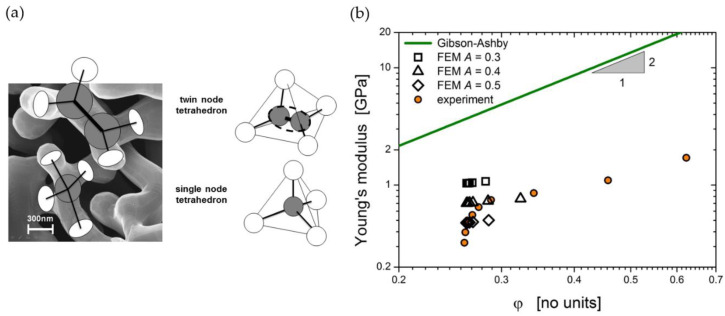
(**a**) The model of np-Au with periodic diamond cells by Huber et al. [89] and (**b**) predicted elastic modulus during elastic–plastic compression for different degrees of randomization. Reprinted from [89] with permission from Elsevier.

Huber et al. have combined FE simulations with the compression testing of mm-sized samples of np-Au [89]. For gaining simultaneous information about the evolution of the macroscopic flow stress and Young’s modulus, the loading history included unload–reload cycles, which were inserted at defined strain increments. Motivated by SEM images, where nodes with three as well as four ligaments were visible, the nanoporous material is modeled as a diamond structure, see Figure 13a.

The geometry is primarily defined by the aspect ratio of the ligaments, r/l, where r and l denote the radius and length of the ligaments, respectively. Additional geometry parameters are the size of the nodal mass, $c_{R}$, inspired by [93] and the degree of randomization of the network, A, perturbing the nodes by a random displacement in space, similar to the structural irregularities previously applied in foam modeling [97]. For the elastic–plastic simulations, isotropic plasticity with linear work hardening was assumed. The FE beam model has been validated with an FE solid model for a selected geometry. The computational efficiency of the FE beam model allowed for an investigation of the structure–properties relationship for a large range of geometries from very thin to very thick ligaments, as well as from perfectly ordered to strongly randomized network structures. The comparison of the results from the FE beam model with the FE solid model revealed that for ligament aspect ratios r/l≳0.1 and similar values of solid fraction, the nodal mass substantially stiffens and strengthens the macroscopic response of the material for which a correction needs to be added to the scaling law. For values below, the Gibson–Ashby scaling law $E=C_{b}\varphi^{2}$ is confirmed in agreement with foams with bending as the dominating deformation mechanism, but the leading constant is reduced to $C_{b}=0.34$ due to the different network structure [89]. 

A major advantage of the FE beam model is the decoupling of the solid fraction (variation of r/l, $c_{R}$) from the network structure (variation of A), which is not trivial to be controlled independently in structures generated by phase-field or kinetic Monte Carlo methods. By variation of the structure randomization, Huber et al. have shown that the degree of structural randomness has a strong impact on the macroscopic stiffness, flow stress, and elastic and plastic Poisson’s ratio [89]. In contrast to low-density foams [24], all properties showed a strong decrease for increasing randomization. For reproducing the absence of lateral strain observed in compression tests, the randomization should be A≳0.3. Tuning the macroscopic Young’s modulus to the data measured from the unloads has suggested a value of A≈0.4, see Figure 13b.

With this structure, the macroscopic flow stress was fitted by tuning the material behavior with two surprising results. First, the yield stress of the solid phase was determined unexpectedly low to a value of $\sigma_{y,s}=67$ MPa for a ligament size of 63 nm. Second, to reproduce the measured rise of the macroscopic flow stress, a work hardening rate of $E_{T,s}=1$ GPa was required, pointing toward dislocation accumulation and increasing dislocation–dislocation interaction as the deformation proceeds. Further, the initial Young’s modulus was found to be a factor of 10 below the Gibson–Ashby. After incorporating the effect of randomization in the computation of the solid fraction of the RVE, the required randomization reduces to a value of A≈0.23 [92]. A method for a nodal correction of the FE beam models has been added by Jiao and Huber, which brought the determined yield stress $\sigma_{y,s}$ in a better agreement with results in literature data [113]. This shows that the nodal correction becomes an important aspect in FE beam modeling of nanoporous metals that have higher solid fractions compared to foams. Therefore, a more general nodal correction has been developed by [114] for the elastic–plastic deformation of parabolic-spherical ligament shapes suggested in [50] that allows the prediction of the stress–strain curves of the structures derived from tomography data with an accuracy of about 20%. 

The conclusion that work hardening should be present during the plastic deformation of np-Au motivated simulations focusing on defect generation and accumulation via MD simulations by Ngô et al. [115]. Following [89], the macroscopic compression test consisted of subsequent loading-unloading cycles. The virtual sample is generated by Monte Carlo simulation, thereby reproducing the interconnected nanoscaled network structure of np-Au with an average ligament diameter of the ~3 nm at ~40 nm edge length of the simulation box and a solid fraction of φ=0.3. The equilibration at 300 K leads to densities of dislocations, stacking faults, and twins that are not negligible. The predicted stress–strain curve shows a very similar behavior to the experiment, although the average ligament size is ~40 nm in the experiment. The plastic deformation induces a progressive increase in the dislocation density and planar fault density adding up to ~4 dislocations per ligament at the end of the deformation. Planar defects are only found in the nodes. Both the experiment and simulations show plastic deformation after unloading in the early stages of ~1% strain—i.e., the onset of yielding happens immediately as load is applied. This is interpreted as a consequence of the distribution of the resolved shear stress originating from the surface-induced prestress in a heterogeneous ligament network. 

The macroscopic Young’s modulus, determined from inserted unloads, show an excellent agreement between the MD simulations and the experiment [115]. At any time during compression, np-Au is much more compliant than predicted by the Gibson–Ashby law. Remarkably, substantial stiffening occurs already at a small plastic strain, while the net surface area is essentially invariant. This observation rules out the surface excess elasticity as a possible explanation, and Ngô et al. attribute the effect to shear instability. In a subsequent work [116], Ngô et al. have compared their results with FE simulations for the very same structure, thereby yielding a stiffness that is a factor of 2.8 higher. It is concluded that the enhanced compliance, as predicted by MD simulations, is expected only at very small ligament diameters d where the surface-induced stress and the ensuing shear strain are high. For ligament sizes ≳20 nm present in the experimental studies, it is argued that the high compliance results exclusively from network geometry effects such as low scaled connectivity density [18,78,79,85], see Section 3.3. 

#### 3.1.3. Macroscopic Tension until Fracture

**Figure 14:**
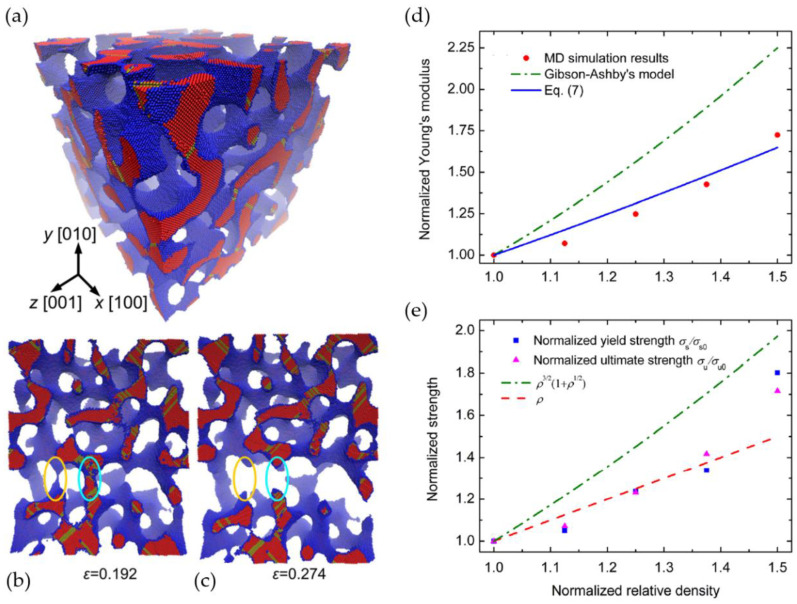
(**a**–**c**) Deformation behavior of np-Au under tension showing pinching-off of ligaments; (**d**,**e**) Comparison of simulation results with the Gibson–Ashby scaling laws and a modified version with a second term accounting for tension along the ligament axis. Reprinted from [117] with permission from Elsevier.

Sun et al. have used this technique to generate two types of samples that have (I) the same ligament diameter of d=3.26 nm with solid fractions ranging from φ=0.24 to 0.36, and (II) samples that have a constant solid fraction of φ=0.30 with ligament diameters varying between d=2.45 and 4.08 nm [117]. All the samples are studied under uniaxial tension, as shown in Figure 14, showing initially a linear slope and a stress decrease after reaching a maximum stress value denoted as ultimate tensile strength $\sigma_{u}$. During the tensile deformation of a sample with φ=0.30 and the ligament size of d=3.26 nm, the fraction of surface atoms and of hexagonal close packed (hcp) atoms increase to ~0.1% and ~1%, respectively, where the latter is attributed to the formation of stacking faults and twin planes. The emission of partial dislocations emitted from free surfaces caused serrations in the stress–strain curves. At applied strains of 23%, the weakest ligaments aligned in loading direction start to rupture, similar to a single crystalline gold nanowire, and ligaments that are initially not perfectly aligned rotate toward the tensile direction, see Figure 14b,c. The yield strength and ultimate tensile strength is found to be effectively dominated by the axial yielding of ligaments—this corresponds to the linear scaling with the solid fraction. In addition, the ultimate tensile strength showed a strong dependence on the average ligament diameter and scaling with an exponent of −0.44 [117]. This is close to the exponent of −0.5 in the Hall–Petch model [112]. 

As a consequence of the alignment of ligaments, Sun et al. have added a second term to the Gibson–Ashby scaling law for the macroscopic Young’s modulus in such a way that $E/E_{s}=C_{2}\left(\varphi^{2}+C_{3}\varphi \right)=C_{b}\varphi^{2}+C_{t}\varphi )$, Figure 14d. In this way, the scaling law also accounts for the effect of ligament tension. Sun et al. have calibrated the leading constants by using the results from the MD simulations, yielding $C_{b}=0.14$ and $C_{t}=0.136$. In combination with the exponents in the scaling law, the equation implies that tension becomes the dominant mechanism for the decreasing solid fraction [117]. 

Winter et al. have looked into the tensile deformation and fracture of np-Al. The phase field method was used to generate six models with a box edge size of 20 nm, a ligament diameter of d=5 nm and solid fractions ranging from φ=0.36 to 0.84 [118]. In addition, four models are generated for a solid fraction of φ=0.4 with the ligament diameters d=3.11–9.88 nm. The stress–strain behavior and failure mechanisms are very similar to those reported by [117]. First of all, Winter et al. have found that the macroscopic Young’s modulus is not affected by the ligament diameter within the studied range, but it progressively decays with increasing temperature and reaches about 50% of its initial value at a temperature of 600 K due to the increased kinetic energy present in the system, thereby allowing for atom de-bonding at a lower level of stress. An attempt to fit the scaling law for Young’s modulus by Sun et al. provided a good agreement for solid fractions φ≳0.45, while for lower solid fractions the fit delivered negative values. To solve this issue, Winter et al. have suggested a modified scaling law of the form $E=92.07\varphi^{3}$ [118].

Assuming a bulk Young’s modulus for the Al solid phase of $E_{s}=71$ GPa, the leading constants determined by Winter et al. are $C_{b}=1.7$ and $C_{t}=-5\times 10^{-3}$. The modified scaling law corresponds, then, to $E/E_{s}=C\varphi^{3}$, where C=1.3. However, according to Gibson and Ashby, no deformation mode exists that can yield an exponent higher than 2 [119]. Therefore, a scaling law that contains an exponent of 3 and a term with a negative leading constant is not very meaningful. The problem is that any scaling law based on the solid fraction as the only independent variable is implicitly based on the supposition that the coordination of the ligament in the nodes remains unchanged. Gibson and Ashby have used a unit cell that has a coordination number of 3, and the solid fraction is tuned by the ligament thickness. We will discuss this in more detail in Section 3.3. What has been missed is the additional effect that is brought into the equation by the connectivity density of the ligament network, which substantially changes along with the varying solid fraction [65]. Further, in contrast to the structure generation with random Gaussian fields, as used by Soyarslan et al. [65], the phase field method used by Winter et al. seems to allow for an independent tuning of the solid fraction and the connectivity density, similar to liquid metal dealloying [78,85]. Therefore, it is not recommended to fit the Gibson–Ashby scaling law to simulation results where the coordination of the ligaments is variable. It can be argued that important information about the statistics of coordination numbers or scaled connectivity densities should be additionally considered, as already pointed out by Saane et al. [15] and later discussed in detail by Huber [80]. 

With respect to the size effect, Winter et al. have determined a dependency of $\sim d^{-0.36}$ [118]. As this is determined for the constant solid fraction of φ=0.4, it can be assumed that this value is determined for the same scaled connectivity density. The larger ligament diameters are close to 10 nm, which, according to [112], marks the beginning of the transition into size-independent yield strength. In contrast to the macroscopic Young’s modulus, the yield stress decreases linearly over temperature to 60% at a temperature of 600 K. 

Winter et al. have determined the toughness from the area under the individual stress–strain curves until reaching the ultimate tensile strength. The fit of the data leads to a scaling of the form $K=170\varphi^{3}$ [118]. Beyond the question, if an investigation of solid fractions of more than 50% is meaningful, see also [65], the brittleness of nanoporous metals under tension is an issue that cannot be ignored. Briot et al. have performed experiments on pseudo single crystalline np-Au tensile tests for ligament sizes ranging from 32 to 60 nm [120]. After multiple loading and unloading segments, the specimen showed no plastic deformation and failed by brittle fracture in the gage section. The determined $K_{IC}=10.4\;{\mathrm{Jm}}^{-2}$ is just about double the value of highly brittle materials. Recently, Badwe et al. have carried out tensile tests for samples with ligament diameters ranging from 1.74 to 742 nm and found a ductile regime for ligaments above about 220 nm. Below this ligament size, the fracture is brittle [49]. As the MD simulations do not predict brittle behavior, it would be helpful to critically evaluate the determined toughness values in comparison with the experimental results. 

#### 3.1.4. Multiaxial Yielding

So far, all micromechanical models use macroscopic compression or tension in accordance with experimental works. However, in experimental set-ups with an indenter or a flat punch, multiaxial deformation is caused by barreling under compression [121,122]. Mangipudi et al. have pursued the first study on the elastic–plastic deformation behavior under multiaxial loading conditions to discover the origin of inconsistencies between the reported values on the plastic Poisson’s ratio $\nu_{p}$ and hardness to yield stress ratios $H/\sigma_{y}$ for different types of experiments and samples [123]. Elastic–plastic multiaxial simulations for the reconstructed np-Au samples and RVEs [18,34] are carried out for plain stress and axisymmetric loading conditions, thereby revealing a coincidence of the normalized yield loci for the two types of structures regardless of their different levels of network connectivity (see Section 3.3). A comparison of the predictions with the Deshpande–Fleck isotropic foam yield criterion shows that this yield criterion does not capture the predicted shape of the yield surface. In regions with high average stress, there are deviations by about 50%. While the yield surface remains convex, cube-like corners appear in the regions of equibiaxial and equitriaxial stress independent of the sign. For the shape parameter of the yield surface, as determined from the FE simulations, the plastic Poisson’s ratio is determined to be $\nu_{p}=0.23$, which is found to be in excellent agreement with the experimental results for a 15 nm ligament diameter of np-Au and also in qualitative agreement with samples of larger ligament sizes found in the literature. Inserting this value in the hardness-to-yield stress ratio versus the $\nu_{p}$ trend from Shaw and Sata [124] yields 2.7, thereby showing remarkable agreement with the experimental result of 2.7 to 2.8. 

### 3.2. Surface Effects

For ligaments approaching the diameters of a few nm, surface effects contribute to the macroscopic response of nanoporous metals. At the surface, physical and chemical effects are strongly interlinked and provide an entry point for the implementation of actuation, sensing, and tunable mechanical properties into a macroscopic material for which a broad background is available from experimental work [125,126,127,128]. Despite the fascination with functional properties, the literature on the micromechanical modeling dealing with such phenomena is scarce. Two main sources of surface effects are considered—surface stress and surface energy—which will be treated separately in the following subsections.

#### 3.2.1. Surface Elasticity

Feng et al. were the first, who predicted the effect of surface elasticity for nanoporous metals with a micromechanical approach [129]. They combined the continuum theory of surface elasticity [130] with the open-pore unit cell [9]. This extends previous work on size-dependent elastic properties of nanosized structural elements, such as plates and beams investigated in [131], among others, by combining the Gibson–Ashby model with a surface layer of uniform thickness, surface Young’s modulus, and surface stress. The elastic stiffness of the unit cell is determined by distinguishing two groups of ligaments that deform either by extension or by bending. In this way, the effect of the surface stress has been incorporated into the equilibrium equation of the nanobeam with surface effects. The effective modulus is predicted as a function of the ligament thickness for the surface elastic modulus ranging from $E_{s}$ = 0 to 50 N/m and the residual surface stress between $f_{0}=0$ and 500 N/m. For a realistic representation of Au crystal surfaces with $E_{s}=6.6$ N/m, $f_{0}=1.4$ N/m [132], Feng et al. could show that the effect of surface stress and surface elasticity has a significant influence only for a ligament thickness below d<1 nm. With increasing ligament thickness, the effect of the surface becomes less important and even becomes negligible for a thickness of d≳10 nm. In all cases, the predicted effective Young’s modulus is increased—i.e., the model predicts a stiffening of the structure due to surface effects [129]. 

In a multi-scale scheme, Saane et al. have used a combined atomistic and continuum approach to study the charge-induced deformation of ordered and disordered np-Au [15]. To this end, the atomistic model was informed from the sub-atomic length scale through the calibration of the atomistic (SEAM) potentials to DFT-obtained surface–stress–charge coefficients. When a negative charge is injected on the metal surface, the electron density increases in the surface layer that is approximately one atom thick. This leads to a smaller equilibrium spacing of the surface atoms, and the developing surface stress leads to compression of the uncharged core region. The FE solid model of the same geometry accounts for the charge-induced reduction by the assignment of an eigen strain in the surface layer that is proportional to the surface charge. The surface is modeled by plate elements with a thickness that corresponds to the thickness of the charged region. For studying the effect on the actuation strain, a charge density of $q_{a}=7.7\;µC/{\mathrm{cm}}^{2}$ was chosen [15]. The approach is applied to structures with increasing complexity. 

**Figure 15:**
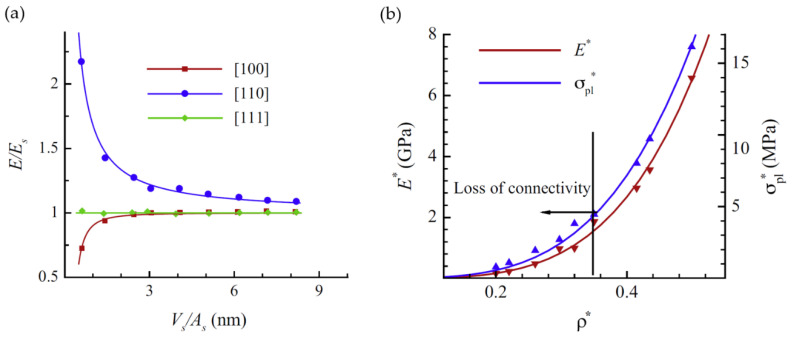
(**a**) Predicted Young’s modulus of nanowires normalized by the bulk modulus for different crystal orientations along the wire axis of width $w=4V_{s}/A_{s}$; (**b**) the scaling behavior of effective mechanical properties with loss of connectivity for solid fractions below around 35% that is caused by voxel erosion. All variables with ${\left(\cdot \right)}^{*}$ denote size-independent values for that specific orientation and density. Reprinted from [15] with permission from Elsevier.

First, atomistic simulations were carried out for wires with [100] and [110] crystal orientation showing good agreement with the predicted actuation strains of the continuum model. As shown in Figure 15a, the sign of Young’s modulus change depends on the orientation of the crystal. It is positive for the [110] and negative for the [111] orientation. Applied to a gyroid structure, the stiffness increases, on average, for decreasing $V_{s}/A_{s}$ ratios, but this effect is much smaller compared to that of the wires. Next, cubic lattices with solid fractions 0.1, 0.2, and 0.3 were tested, showing larger strains for lower solid fractions. Very similar to the results of Feng et al. [129] for decreasing ligament thicknesses, the predicted curves show a rapid increase in the actuation strain for volume-to-surface ratios $V_{s}/A_{s}$ approaching very small values, while for $V_{s}/A_{s}≳10$, the effect becomes negligible. The effect also increases with decreasing solid fractions, but the strain was below the individual nanowire even for the low density lattice due to the nodes/junctions that have no free surface [15].

Moving forward, gyroids with different relative densities, crystal orientations, and volume-to-surface ratios $V_{s}/A_{s}$ were generated. For this structure, the effect of relative density and crystal orientation is found to be much larger than for $V_{s}/A_{s}$, where the effect quickly dies out and converges to the bulk. The authors trace this back to the variation of the crystal orientations of the struts and the curved surfaces in the gyroid—this weakens the stiffening by nonlinear elastic effects. The results for the continuum model nicely follow the Gibson–Ashby scaling laws for Young’s modulus and yield stress of the form $E/E_{s}=0.84\varphi^{2}$ and $\sigma_{y}/\sigma_{y,s}=0.55\varphi^{3/2}$, respectively. With regard to the actuation strain of the gyroid, no effect in the relative density is found, whereas the $V_{s}/A_{s}$ dependency nicely follows a power-law with the exponent −1. Compared to the cubic structure, the magnitude of the actuation decreases by about 25% [15].

For the np-Au model, a real morphological model is obtained by means of FIB-SEM nanotomography on the dealloyed samples published in [18]. Samples with relative densities between 0.2 and 0.5 were generated from the original voxel image with φ=0.35 by three-dimensional morphological image dilation or erosion. The observed unusual scaling behavior $E/E_{s}=1.33\varphi^{4}$ and $\sigma_{y}/\sigma_{y,s}=1.05\varphi^{3.5}$ is observed, as seen in Figure 15b.

The high scaling exponents are be traced back to a loss of connectivity that goes along with decreasing density, resulting in a much stronger decrease in stiffness and strength. Further, for densities equal to and above 0.35, the dependence of the relative density changes from a quadratic into a linear dependency—i.e., $\varphi \propto {\left(r/l\right)}^{2}\to \varphi \propto \left(r/l\right)$, as found by Liu and Antoniou [93], explaining the high exponent in the scaling law for the yield stress. Regarding the size-dependent actuation behavior, the power law scaling with an exponent of −1 is confirmed also for np-Au with a magnitude that is only 8% below the value of the cubic structure. Taking the limitation by the yield stress into consideration, Saane et al. have determined the work density for the three structures, with cubic yielding the highest and np-Au the lowest values. This shows that the work density increases with increasing order and connectivity. Further, the optimal actuation performance is achieved for the largest surface to volume ratios, while the porosity should be minimized [15]. 

Elsner et al. have determined the surface excess elastic parameters as a quantitative measure of the local elastic response and possible key factor regarding the origin of the size-dependent effective properties of nanomaterials [133]. The authors have derived closed-form expressions for the effective stiffness of a nanowire under different loading conditions, supported by the direct insertion of the DFT-derived surface elastic parameters. By combining a continuum analysis with a DFT study, the impact of surface excess elasticity on the effective stiffness of nanoscale structures was discussed for Au(111) and (001). By inserting the DFT-based surface excess elastic parameters in the continuum model, it is shown that the surface excess elasticity has the strongest effect on the torsion stiffness, followed by bending and axial tension. In agreement to the works of [15,129], only extremely small diameters below 10 nm lead to a detectable effect in the effective elastic response [133]. 

However, Elsner et al. have found “positive- as well as negative-valued surface excess elastic parameters. In combination, their impact on the effective elastic response of nanowires under various deformation modes is an enhanced compliance at small size” [133]. This contradicts the stiffness increase predicted by [15,129] and implies that the assumptions made for the implementation of surface elasticity in these micromechanical models are oversimplifying the problem. As shown by Michl et al. with ab inito simulations, the situation is far more complex in such a way that a physically sound integration of the surface stress–charge response would require more attention [134]. With respect to the surface excess elastic parameters, Elsner et al. show that the empirical potentials of previous studies are limited in their transferability and that the more rigorous approach by using DFT has a considerably more substantiated basis. Further, they point out that the different conventions of defining stress and strain measures in continuum theory and atomistic modeling should be carefully considered [133]. In view of this effort and with the insight that the impact of the surface excess elasticity can be excluded as the origin of the experimentally observed size-dependent properties of nanoporous metals, it is justifiable to ignore this effect in micromechanical models.

#### 3.2.2. Surface Energy

Mameka et al. have shown experimentally that plastic yielding is not prompted by surface stress. Instead, the measured surface tension as the second capillary parameter has been identified as the relevant driving force [128]. It can be understood as a measure for the surface energy that works against an increase of the surface area by plastic deformation and, therefore, represents an alternative mechanism impeding yielding under tension and assisting yielding in compression. Lührs et al. have investigated the role of the surface during plastic deformation, thereby verifying the surface-induced tension–compression asymmetry predicted by [112] in an experiment that uses electro-chemical cycles during ongoing compression deformation [135]. The difficulty in the experimental proof of tension–compression asymmetry was the brittleness of np-Au that limits the experiment to elastic deformation. Therefore, Lührs et al. have exploited that network structures respond to macroscopic compression by transverse plastic strain, revealing the signature of tension–compression asymmetry. The surface energies for the applied potentials of 1.5 V (one monolayer of oxygen species) and 0.8 V (clean surface) were determined by electrochemical characterization to $\gamma_{1.5V}=0.4\;{J/m}^{2}$ (surface energy “on”) and $\gamma_{zc}=1.5\;{J/m}^{2}$ (surface energy “off”), respectively. Switching the potential allows the switching on or off the surface energy during the ongoing compression experiment. By continuous measurement of the plastic Poisson’s ratio for np-Au (d=40 and 70 nm; φ=0.25 and 0.28), the signature of the tension–compression asymmetry could be measured as visible jumps in the plastic Poisson’s ratio of ~0.05 [135]. 

**Figure 16:**
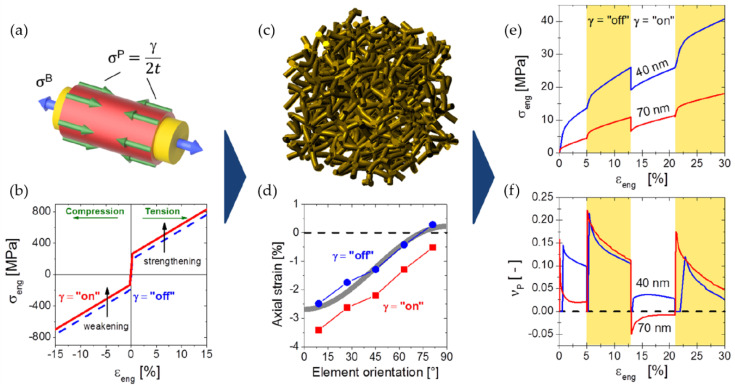
(**a**) Pre-stressed surface modeled as pipe elements inducing (**b**) the tension–compression asymmetry in the ligaments. (**c**) The micromechanical model for the integration of surface energy in a randomized diamond structure with elastic–plastic cylindrical ligaments. (**d**) The orientation of ligaments explains the origin of the mechanism that leads to the jumps in the plastic Poisson’s ratio. The model reproduces (**e**) switchable flow stress, and (**f**) jumps in plastic Poisson’s ratio. Reprinted from [135] in accordance with ACS Author Choice License.

For a detailed understanding of the underlying mechanism, Lührs et al. have established a micromechanical model by extending the RVE compromising a randomized diamond structure according to [89,92] by using a pipe/core configuration [135]. As depicted in Figure 16a,c, the core of the ligaments is modeled with cylindrical beam elements and elastic–plastic deformation behavior, whereas pipe elements add the surface energy in the form of a rubber-like material of a very low Young’s modulus. The pipe elements are pre-stressed in tension through a thermal shrinkage before the RVE is loaded in compression. In contrast to surface excess elasticity, which acts symmetrically for applied elastic strains, the low modulus of the pipe elements keeps the stress level during the elastic–plastic deformation of the core approximately constant, thereby mimicking a constant surface energy per surface area. 

Owing to the constant tensile stress in the surface, it adds constant compression stress to the core independent of its deformation. This configuration works against the plastic elongation of a ligament under tension, whereas it promotes the plastic flow of the core during compression. By switching the temperature, the simulated effect of the surface energy can be switched on and off at any time during the macroscopic compression of the RVE. In this way, the experimentally measured effect in macroscopic strength and plastic Poisson’s ratio could be reproduced in a semi-quantitative way, see Figure 16e,f. The direction of deformation depends on the orientation of individual ligaments relative to the loading axis. Those ligaments which are aligned with the loading axis entail local compression, whereas those oriented perpendicular to the loading axis are loaded in tension, see Figure 16d. Switching the surface energy on supports the compression in the loading direction and reduces the macroscopic strength (Figure 16e), whereas it hinders the transverse expansion and reduces the plastic Poisson’s ratio (Figure 16f). This effect increases with decreasing ligament size and is shown to be fully reversible in the experiment and in the model [135].

### 3.3. Topology

#### 3.3.1. Scaled Genus/Connectivity Density

Mangipudi et al. have continued to work on a more detailed quantitative morphological and topological characterization [18], as suggested in [15]. This represents a crucial advancement beyond the discussion of the mechanical response of bicontinuous network structures solely based on the relative density as independent variable, thereby adding a new quality in the understanding of the structure–properties relationship. To this end, Mangipudi et al. have considered structures with a constant relative density of φ~0.32, namely (i) tomographic reconstructions of two np-Au samples, (ii) a spinodal structure, and (iii) a gyroid structure. The elastic–plastic deformation behavior under macroscopic compression was simulated by FEM, assuming isotropic plasticity with a flow stress of 750 MPa. Among all structures, the gyroid turned out to be the stiffest and the strongest, whereas the spinodal structure showed the lowest stiffness and strength. The two stress–strain curves for the reconstructed np-Au fell in between these two and agreed very well with each other. The results for the gyroid could be explained based on its highly regular lattice. However, the np-Au reconstructions and the spinodal structure appeared to be similarly randomized, yet the mechanical properties were strongly different. The detailed topological examination revealed that in np-Au more than 80% of the nodes are triple junctions, while less than 20% are four-fold connected. The same composition was determined for the spinodal structures. Therefore, both structures represent randomized versions of an unequal combination of gyroid and diamond structures, and the nodal connectivity could not explain the observed differences in the mechanical properties. Furthermore, structural anisotropy, tortuosity, and non-uniform cross-sections characterized by interfacial shape distribution (ISD) could be ruled out, as all these effects contribute only a small fraction [18]. 

**Figure 17:**
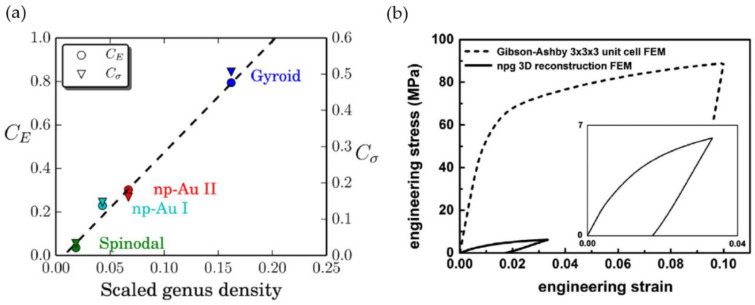
(**a**) Leading constants in the Gibson–Ashby scaling laws predicted for constant solid fractions depend linearly on the scaled genus density, reprinted from [18] with permission of Elsevier and (**b**) the stress–strain response of the np-Au reconstruction compared to the Gibson–Ashby model of same solid fraction [19], reprinted by permission of Taylor & Francis Ltd.

In contrast, the scaled genus density $\bar{g}$ as another topological parameter characterizing the overall connectivity showed very different values for the three structures. With this finding, Mangipudi et al. have been able to find a linear correlation between the scaled genus density, describing the topology and the pre-factors of the Gibson–Ashby scaling laws of the form $C_{E}=5\bar{g}$ and $C_{\sigma }=3\bar{g}$, as shown in Figure 17a. They conclude that the foam density and the foam topology effects can be separated. Mangipudi et al. also point out that the genus increases only slowly with the number of cells and that the cutting of the sample at boundaries lowers the genus. Therefore, a more appropriate scaling by defining a “genus per cell” was suggested as a preferable solution, but such a representative cell is difficult to be identified unambiguously in np-Au—this necessitates further studies to capture the connectivity into scaling laws in a more general form [18].

In parallel and very close to the work of Mangipudi et al., Hu et al. present a morphological and topological analysis of a 3D reconstruction of np-Au that was coarsened from d = 26 nm to a mean ligament thickness of d = 420 nm with a relative density evolving from φ=0.3 to 0.34 [19]. As the first part of this investigation, the required RVE size was addressed for the most coarsened structure by the analysis of the scatter of different characteristic parameters as a function of the cubic length. From plotting the ligament volume fraction vs. the cubic length of a total of six regions, Hu et al. find that the results converge around $L_{RVE}=$6 µm, which corresponds to a ratio of $L_{RVE}/d\sim 15$. For sufficiently large RVEs, Hu et al. have studied the geometric self-similarity and confirmed this for all ligament sizes in terms of the distribution of the ligament thickness and the scaled connectivity density. Together with the good agreement of the scaled ligament size distributions, this served as evidence of the self-similarity of the np-Au samples [19]. Later, this was confirmed by kinetic Monte Carlo simulations performed by Li et al., who could show that structures with φ≥0.3 maintain their connectivity during coarsening, while those with φ<0.3 become more and more disconnected as the coarsening proceeds [47].

Hu et al. have also compared the elastic–plastic response of the np-Au RVE under macroscopic compression with the one predicted for the Gibson–Ashby structure of the same relative density, which has given a much stiffer and stronger response (Figure 17b). It is argued that the source of this discrepancy is related to the distribution of the load-bearing solid phase. While in the Gibson–Ashby model all of the network structure contributes to closed rings that bear load, many “dangling” ligaments are found in the np-Au structure. These singly connected ligaments do not contribute to the load transfer and represent dead mass. Motivated by this observation, the authors suggest considering only the volume fraction of the solid volume that contributes to closed rings within the np-Au network. After skeletonization and pruning of dangling ligaments, Hu et al. find that only 10–14%, instead of the initial 30%, volume fraction forms the ring network. Inserting this reduced relative density in the Gibson–Ashby scaling law brings the predicted macroscopic Young’s modulus down from 8 to 0.8–1.7 GPa, which is much closer to the value of 400 MPa, as predicted by the FE solid model generated from the FIB-SEM nanotomography [19]. 

Regarding the structure–property relationship, the independently published works by [18] and by [19] reached the same conclusion, emphasizing the connectivity as a key parameter for the structure–property relationship. While Mangipudi et al. have found a proportionality between the pre-factors of the Gibson–Ashby scaling laws and the scaled genus density, Hu et al. suggest taking only the volume fraction of the effective load-bearing ring structure into account. Knowing that the genus is counting the number of non-redundant closed-loop paths by which all regions inside the structure can be inspected, the two approaches are qualitatively equivalent. 

#### 3.3.2. Percolation

As shown by Sahimi [136] and Kováčik [137], the macroscopic mechanical behavior of network structures and porous materials can be modeled by means of percolation theory. A thorough study for various kinds of ordered and random foams was presented by Roberts and Garboczi, who have suggested a three- or four-parameter relation to describe the macroscopic Young’s modulus for the open-cell tessellation in the full density range by a scaling law of the form $E/E_{s}={\left[\left(\varphi -\varphi_{c}\right)/\left(1-\varphi_{c}\right)\right]}^{f}$ [20]. They point out that the parameters and f=2.12 and $\varphi_{c}=-0.0056$ that fitted their simulation results are not the conventional exponent and percolation threshold. 

**Figure 18:**
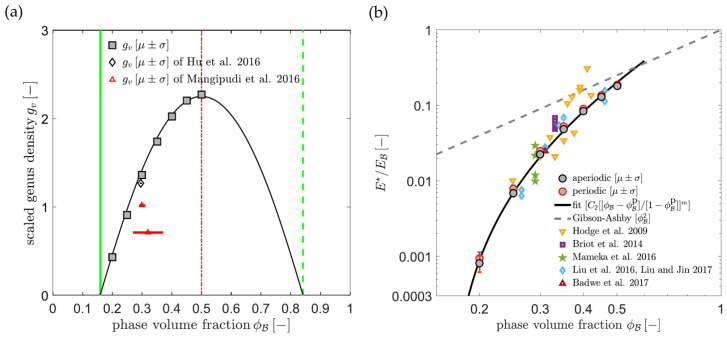
(**a**) Scaled genus density as a function of the solid fraction for structures generated from random Gaussian fields and (**b**) predicted macroscopic Young’s modulus compared to experimental results from the literature. Reprinted from [65] with permission of Elsevier.

Continuing the discussion along the path of random network structures on the upper right of Figure 11, Soyarslan et al. have used structures generated with Gaussian random fields over a large range of relative densities from φ=0.0 to 1.0 for a systematic investigation [65]. Despite the random nature of the resulting microstructure, this approach produces periodic structures by superimposing a finite number of plane standing waves of the same wavelength, but a different phase shift and orientation. Concerning the morphological characterization, a characteristic structure size $\tilde{L}$ is derived as the mean distance between the local centers of the solid or the pore space. Together with the edge length of the unit cell, this measure allows the computation of the number of microstructural elements per unit cell volume. As shown in Figure 18a four types of microstructures evolve, which are (i) convex ellipsoids of the disconnected solid phase that form for relative densities below the threshold for percolation to cluster transition $\varphi <\varphi_{c}$ and (ii) thin and long ligaments with a morphology similar to those found in np-Au for $\varphi_{c}<\varphi <0.5$. Owing to the symmetry at φ=0.5 that equally splits solid and pore space in the RVE, the other two microstructures correspond to the previous two microstructures when we consider the pore space. Therefore, for $\varphi >1-\varphi_{c}$, isolated pores are formed [65]. 

Soyarslan et al. have derived a relationship for the scaled genus density as a function of the phase volume fraction φ [65], which has also shown good agreement with the data point measured by [19] for np-Au of φ=0.33, whereas the values determined by [18] are significantly lower (Figure 18a). For solid fractions equaling the percolation threshold $\varphi_{c}=0.159$, the scaled genus density is zero and rises to a maximum value of 2.3 at a solid fraction of φ=0.5. For a sufficiently large RVE, Soyarslan et al. have determined the macroscopic Young’s modulus and Poisson’s ratio as a function of the solid fraction. They show that a volume size of 12 wavelengths delivers converged values of the effective elastic properties. For aperiodic structures, 10,000 waves are required for producing sufficient isotropy with aperiodic structures, whereas a comparably small number of 96 waves satisfying $H=\sqrt{146}$ provide an identical quality for periodic structures. The predicted data for the macroscopic Young’s modulus can be described by a modified version of the Roberts-Garboczi scaling law [20] of the form:

(3) $$\frac{E}{E_{s}}=C_{2}{\left(\frac{\varphi -\varphi_{c}}{1-\varphi_{c}}\right)}^{f}$$

where $C_{2}=2.03$ and f=2.56. In contrast to Roberts and Garboczi, here $\varphi_{c}$ = 0.159 is the percolation threshold at which the network loses its connectivity—i.e., for lower relative densities, there exists no load transfer path from the top to the bottom face. Figure 18b shows that the fit to the predicted data points Equation (3) matches well with data collected from various sources in the literature, particularly for solid fractions below φ=0.35. In addition to Young’s modulus, an empirical law could be fitted to the FE results for Poisson’s ratio of the form $\nu /\nu_{s}=D_{1}\log\;\varphi +D_{2}$ with $D_{1}=0.116$ and $D_{2}=0.363$ [65].

In essence, Soyarslan et al. have concluded that the random-field microstructure is found to be a rather accurate representation of the microstructure of np-Au. Evidently, there is a link between the connectivity and the stiffness of np-Au. It is shown that reducing the solid fraction of np-Au moves its network structure toward the percolation-to-cluster transition by reducing its topological genus. This is also seen as an important element during the coarsening of spinodal-like structures. Soyarslan et al. point out that this phenomenon may hold the key for understanding the systematic deviation between experimental np-Au stiffness data and the classic foam scaling relations [65]. In view of the other literature discussed before in this review, it can be added that this also holds in the same way in terms of the unusual high exponents in scaling laws, as observed in [118] and [18]. The simplicity of the random-field microstructure is of great advantage for most applications because the structure generation has only one free parameter, which is the solid fraction φ. This comes at the cost that this model does not allow to vary the topology independent of the solid fraction, as this is pointed out by Soyarslan et al. who “limit the comparison between experiment and the connectivity or stiffness data of our model to experimental samples that were in their as-prepared state. Coarsened samples are excluded from the comparison because of their likely lesser-scaled genus” [65].

#### 3.3.3. Evolution of Network Connectivity during Coarsening

In addition to self-similarity, discussed in Section 2.4, another debate among experimentalists was concerned with the evolution of the network connectivity during the coarsening of np-Au with φ≳0.3. While Hu et al. have reported geometric similarity along with constant-scaled connectivity density during the coarsening of their np-Au samples [19], Liu et al. could provide evidence for a reduction of the network connectivity during the coarsening process at φ=0.26 [78]. In a further work [85], Liu and Jin could show that the loss of the macroscopic Young’s modulus is enhanced for the samples that are prepared with a lower relative density, while for samples with φ=0.46, no change in macroscopic Young’s modulus could be observed. All the samples were coarsened from initially 4 up to 600 nm in the ligament diameter [85], see also [6]. 

Geslin et al. have looked into the coarsening process using a phase field model for surface diffusion that is well suited to simulate the evolution of nanoporous materials [138]. The simulations were limited to samples in the higher range of solid fractions from φ=0.375 to 0.5. For all samples, the simulation results show perfectly comparable ISD plots and nearly constant scaled connectivity densities over the coarsening time. This behavior has been experimentally confirmed with the microstructures of FeCr samples obtained using X-ray tomography, and it is shown that all coarsened in a self-similar manner [138]. For the relevant range of solid fractions, this is in agreement with Liu and Jin [85], who, however, have observed a moderate loss of connectivity for np-Au samples with φ=0.35 and a rapid loss of connectivity for φ=0.26. The first work that provided the full picture on the coarsening evolution over the whole range of solid fractions has been carried out by Li et al. [47]. Although no mechanical properties have been computed for these structures, the evolution of the scaled genus density g shows two very different behaviors. For solid fractions φ≥0.3, a constant g indicates self-similarity during coarsening, whereas below this value, g tends to decline: The more φ approaches the percolation threshold $\varphi_{c}=0.16$, the faster the loss of scaled connectivity density [47], see Figure 8 in Section 2.4.4. Consequently, coarsening is not generally self-similar. 

For implementing the loss of connectivity in a micromechanical model, we can interpret the pinching-off mechanism during coarsening as a random fracture of connections in the network structure. Here attention should be directed to Sieradzki and Li, who have investigated the fracture behavior of a solid with random porosity [139]. Within samples modeling a 2D triangular network of ligaments, voids were randomly cut and thereby a fraction of the ligaments has been mechanically deactivated. Double logarithmic plots of the measured data for the normalized Young’s modulus $E/E_{0}$ versus the porosity $p-p_{c}$ show a linear dependency that could be fitted to the percolation function of the form $E/E_{0}\sim \;{\left(p-p_{c}\right)}^{f}$, where $p_{c}\approx 0.63$ is the percolation threshold. The elasticity exponent f=3.1 has been found to be close to the value of 3.5 from theoretical studies [139]. A similar scaling relation has been found for the normalized fracture stress that follows a different slope of f=1.7. Reviewing the literature on 3D networks, Kováčik finds a broader distribution of percolation thresholds and exponents [137]. In the context of 3D percolation theory, the model assumes a value f=3.75 for a cluster dominated by bond-bending forces when the dimension of the system tends to infinity for all dimensions [136] (p. 185). Kováčik concludes that smaller samples and sample preparation can have a strong influence on the value of f, leading to lower values close to f=1.2. Furthermore, the percolation threshold for the porosity from different sources strongly varies from $p_{c}=0.06$ to 0.6. 

**Figure 19:**
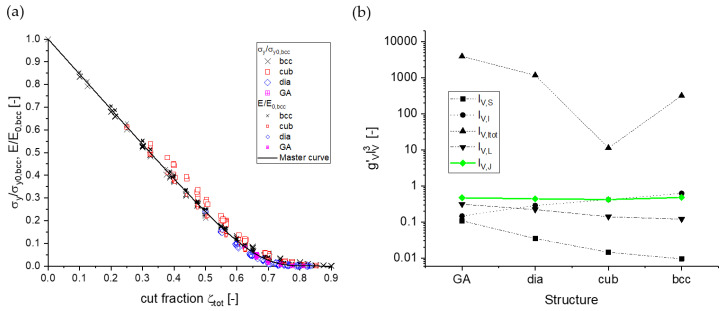
(**a**) The master curve for the macroscopic Young’s modulus and yield stress as a function of the cut fraction (data shown combined supplementary data for both properties provided [80], Data sheet 2) and (**b**) variation of scaled genus density normalized with different characteristic lengths plotted against structures with increasing coordination number. Reprinted from [80], published under Creative Commons Attribution License (CC BY 4.0), own work.

To investigate the percolation behavior of 3D networks, Huber has generated RVEs with varying initial connectivity in the form of bcc, cubic, diamond, and Gibson–Ashby periodic cell structures [80]. For these structures, the ligament coordination numbers are z= 8, 6, 4, and 3. By the random cutting of ligaments with a cut fraction ζ, the load-bearing solid fraction has been continuously decreased until the percolation threshold $\zeta_{c}$ is reached. As expected, a structure with a lower initial coordination number shows a faster decay in the mechanical properties that leads to an earlier percolation-to-cluster transition. At the same time, while the percolation threshold $\zeta_{c}$ decreases with the decreasing coordination number z, the exponent f also increases. By scaling of the datasets for the different structures, Huber could show that the reduction in both mechanical properties as a function of the cut fraction ζ follows the very same master curve, independent of the structure used. This master curve, shown in Figure 19a (solid black curve), consists of initially linear decay with a sigmoidal transition and horizontal tangent at the percolation threshold, given as:

(4) $$\frac{E}{E_{0,bcc}}=\tilde{E}\left(\zeta_{tot}\right)=1-a_{0}\zeta_{tot}+\frac{a_{1}}{\left[1+\exp\left(-a_{2}\left(\zeta_{tot}-\zeta_{c}\right)\right)\right]},\;0\leq \zeta_{tot}\leq \zeta_{c}$$

In this equation, $\zeta_{tot}$ is the total cut fraction relative to a fully connected bcc structure with a coordination of z=8, and $\zeta_{c}=0.822$ is the percolation threshold for bcc. For a given initial rate of decay $a_{0}$, the other two parameters $a_{1}$ and $a_{2}$, can be computed as a function of $a_{0}$ and $\zeta_{c}$. Therefore, the observed variation of the exponent f, which has different interpretations in the literature, can be simply traced back to the variation in the curvature of the current segment of the master curve that applies to the structure under investigation [80]. The yield stress for cubic structures, shown in Figure 19a, deviate to higher values. This structure is the only one that requires a rotation and subsequent cutting to a cubic RVE to induce bending deformation. Therefore, ligaments at the boundaries are shorter and likely show a higher level of resistance against plastic deformation [80].

Further, based on the large variety of structures under investigation, Huber could also show that all three considered topological parameters, namely the average coordination number $\bar{z}$, the total cut fraction $\zeta_{tot}$, and the scaled genus density g, are all linearly dependent on each other [80]. The requirement for this is that each has to be scaled appropriately for ensuring transferability among the structures that are not geometrically similar. Concerning the scaled genus density, a fingerprint of various possible characteristic lengths can be computed, as shown in Figure 19b, from which it becomes evident that only one type of scaling leads to a constant value. This scaling corresponds to the genus normalized by the number of junctions in the fully connected structure—i.e., the division of two numbers where the second one can hardly be determined when the fully connected structure is unknown in the experiment. Therefore, owing to the linear relationship of the three topological parameters, a solution is presented that accesses the problem via the average coordination number which can be determined from the statistical information of detectable junctions. Another consequence is that values for the scaled genus density that are derived with other normalizations, such as $S_{V}^{-1}$ or 〈d〉, do allow only for a comparison within structures that are geometrically similar and cannot be transferred to other structures [80]. 

As a major outcome, a scaling law is proposed that consists of a multiplicative decomposition for the mechanical properties of the form $E={\hat{E}}_{0}\left(\varphi \right){\hat{E}}_{c}\left(\zeta \right)$ [80]. This equation includes the effect of geometry as the first term. The dependency on the solid fraction φ, usually given as a Gibson–Ashby scaling law, can be extended by incorporating a more detailed description of the ligament shape according to [50,53]. The second term integrates the effect of the topology, where the cut fraction ζ can be replaced by any of the other topology parameters $\bar{z}$ or g. Extensions that incorporate other nonlinear independent topology parameters are also possible. Independent of the other, each of the two terms can bring the macroscopic Young’s modulus down to zero. Reducing the solid fraction at constant connectivity leads to the thinning of the ligaments, whereas reducing the connectivity by fracturing ligaments at a constant solid fraction decreases the effective solid fraction according to [85] or reduces the number of load-bearing rings [19,79]. 

By applying the master curve for the macroscopic Young’s modulus to the data in [85], a surprisingly low average coordination number of $\bar{z}\approx 2.0$ results for the samples of lower solid fractions [80]. This value is very close to the percolation-to-cluster transition and explains consistently with the concept of effective solid fraction of Liu and Jin [85] the extremely low stiffness that has been measured for the coarsened samples. 

**Figure 20:**
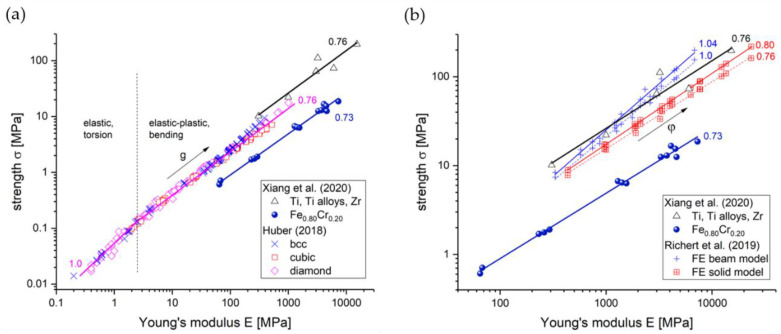
Comparison of the data published by Xiang et al. [14] with the data from (**a**) Huber [80], Supplementary material, Data sheet 2, and (**b**) comparison with the data from Richert et al. [53], Supplementary material, Tables S2 and S4 (thin dashed lines correspond to yield stress, thin solid lines are unpublished data and correspond to flow stress at 10% plastic strain). Slopes entered as numbers in the plots correspond to exponents $n_{\sigma }/n_{E}$ in the dependency $\sigma \sim E^{n_{\sigma }/n_{E}}$.

The master curve Equation (4) serves commonly for modulus and strength [80], which implies a proportionality σ~E for a continuous reduction of the connectivity density. This inspired Xiang et al. to prepare a comprehensive set of nanoporous samples by dealloying of ${\left({\mathrm{Fe}}_{0.80}{\mathrm{Cr}}_{0.20}\right)}_{x}{\mathrm{Ni}}_{1-x}$ in liquid Mg [14]. The samples of the same ligament size around 4 µm and very similar size distribution allow the comparison between Young’s modulus and flow stress over a large range of solid fractions from φ=0.2 to 0.45. These experiments allow for several important observations. First of all, it was experimentally shown that the two coarsening behaviors resemble two different slopes in log mechanical properties vs. log solid fraction plots, intersecting at φ≈0.35. Second, a log(σ)−log(E) plot leads to a slope of 0.73—i.e., $\sigma \sim E^{3/4}$, which is in line with the Gibson–Ashby scaling laws, whereas it contradicts σ~E for simulations with varying connectivity [14]. The comparison of the data from both papers in the form of a log(σ)−log(E) plot in Figure 20a reveals that it can make a big difference of how the data has been analyzed. While plotting $E/E_{0,bcc}$ and $\sigma_{y}/\sigma_{y0,bcc}$ versus ζ in Figure 19a suggests σ~E as a good approximation over all data, the log(σ)-log(E) plot of the same data in Figure 20a nicely reproduces the slope of 0.76, as found by Xiang et al in the higher connectivity range. For very low connectivity values, the exponent changes toward 1.0, indicating a change in the deformation mechanism. This can be explained as follows: When most connections are broken, the few remaining load paths can be interpreted as long ligaments with very low r/l ratios, and likely also with a high level of tortuosity, thereby turning them into elastic springs. Therefore, two mechanisms are likely to be responsible for the change in the exponent: (i) the ligaments deform purely elastic or (ii) the deformation mode changes from bending to torsion [102]. In both cases, the consequence is that σ~E at very low connectivity densities.

It should also be noted that the results presented in [80] rely on efficient FE beam models, which allow for the study of a large number of bigger models needed for introducing a larger number of cuts. However, FE beam models have limitations concerning their prediction accuracy. Foams with low solid fractions φ<0.1 require a compensation of the excess volume due to the overlap of ligaments in the nodes [22]. For higher solid fractions, as present in nanoporous metals, a nodal correction is required that compensates a loss in stiffness and strength by further stiffening and strengthening the nodal region [89,92,113]. This underlying loss is caused by the beam model representation of the nodal mass as a 0-dimensional point compared to the massive nodes in a solid model. From [53], Figure 5 we learn that the loss in stiffness considerably differs from that in the strength if we plot the data versus the solid fraction. This is owning to the locking of the FE beam elements against localized plastic deformation in the massive nodes—this insight leads to the development of a more general nodal correction for parabolic ligament shapes [114]. 

To better understand the impact of this phenomenon in view of the discussion brought up in [14], we plot the data from [53], Figure 5 together with those from [14], Figure 4 in the log(σ)−log(E) plot shown in Figure 20b. The plot confirms a higher slope of the uncorrected FE beam model compared to the FE solid model results which stems from the different influences of the nodal mass on the macroscopic modulus and strength. Moreover, the slope of the FE solid model reproduces the exponent of ~0.75 in line with the experimental data presented in [14] remarkably well. A slightly higher value of 0.8 results in case of the FE solid model, if we use the flow stress measured at 10% plastic strain. From combining the outcome of both plots, it becomes obvious that one should be careful with the interpretation of exponents. First of all, the type of model that is used can obviously bias the translation of structural parameters into different macroscopic properties in different ways. Therefore, the incorporation of a nodal correction that accounts for the individual ligament shape in FE beam models, as proposed in [114], is a necessity for further work on the structure–properties relationships with predictive capability. Similarly, for the interpretation of the experimental data with Equations (1) and (2), we should keep in mind that for φ>0.3 the free length of the ligament becomes less than the ligament thickness and that inaccuracies associated with the volumes of the nodes become severe [9].

Independent of this, the analysis of the data in the form of log–log plots is very convenient for the identification of nonlinear relationships, but one has to be aware that they put much more weight on the lower orders of magnitude. Here we look more closely at the structure–properties relationship where the network has a low solid fraction or connectivity. This is particularly helpful if we are interested in highly compliant structures that are close to the percolation threshold. If the material design aims for any high load-bearing capacity, it would be more appropriate to use linear plots in such a way that the focus of the prediction accuracy and the subsequent analysis are more on the high-stiffness and high-strength data. The decision on how we want to weight the data is independent of using simple plotting or more advanced techniques such as machine learning. Consequently, the best choice of feeding the data into the analysis depends on the objective and requires a certain level of experience. 

### 3.4. Concluding Remarks on Micromechanical Modeling

The reviewed literature on the micromechanical modeling of nanoporous metals shows that two major challenges have drawn the attention of researchers. First, the representation of realistic microstructures and the translation of the complex deformation into macroscopic stress–strain behavior are commonly addressed by the MD and FEM communities. This supports the isolated study of selected structural and material parameters as well as the interpretation of the phenomena observed in experiments. Second, on a higher level, the structure–properties relationships are analyzed, and the existing knowledge is substantially extended. Here FE simulations provide a higher degree of flexibility in the independent parameterization of different structural descriptors, particularly when working with FE beam models that are also highly efficient. These advantages come at the cost of limitations in the prediction accuracy, which can only be avoided by investing substantial work in the development of properly validated nodal corrections [113,114].

In view of the possible effect of facet formation discussed in Section 2.5, Li et al. also looked into the impact of faceting and roughness, which preferably occurred at 900 K and 1800 K, respectively [47]. The authors concluded that the coarsening kinetics are independent of the degree of faceting as there are no qualitative changes in the coarsening behavior between the two temperatures. Concerning the evolution of the ligament shape, the visual inspection of Figures 1 and 2 in [47] seem to support the hypothesis that there is a trend towards more straight and cylindrical ligaments with ongoing coarsening. A detailed analysis of the structures provided in [47] with the approach of Richert et al. [50,53] would provide a reliable basis for a quantitative evaluation.

Surprisingly, little work went into constitutive laws. So far, a lot has been done on elasticity, and only a few works incorporate excess surface elasticity or surface energy in the micromechanical models. The common conclusion is that the effect of excess surface elasticity can be neglected, whereas surface energy has a strong effect on the plastic deformation behavior of the 3D network. Further, plasticity is usually modeled with linear isotropic hardening. Gnegel et al. should be mentioned here as the first work, where, in addition to the behavior of the solid gold, the properties of a polymer coating was also calibrated by fitting an FCM model to experimental data without as well as with coating [72]. Nonlinearities visible in the macroscopic stress–strain response are so far attributed to the deformation of the random 3D network. An interpretation of experimental curves in terms of nonlinearities in the material behavior is very difficult. This would require modelling of the structure of the sample under investigation either directly from the tomography, or ensuring that it is representative in terms of the relevant structural descriptors. With respect to the latter, there is still a long way to go, as discussed in Section 2. 

To date, even well-known experimental effects are so far mostly untouched, among which the size effect [12,13] is the most prominent one. Lacking constitutive models that integrate the size effect in a physically sound manner and allow for reproducing the effects as they are observed in experiments, the common approach is to identify the stress–strain behavior of the solid phase for the given ligament size of a sample. In view of what we discussed in terms of thickness measurement in Section 2, it becomes obvious that there is no such thing as a single ligament size, even if we assume that we could properly measure it. 

**Figure 21:**
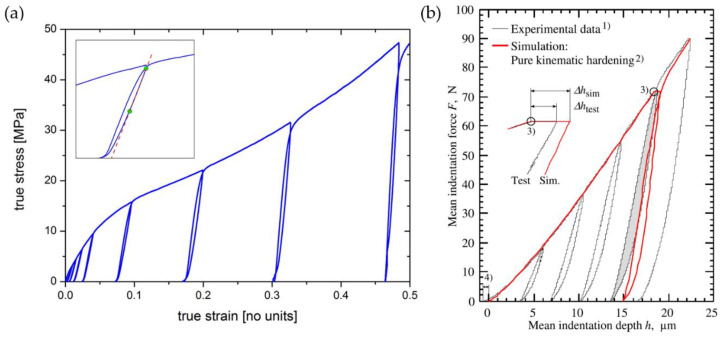
Material response for compression with inserted unloading–reloading cycles: (**a**) Macroscopic stress–strain curves measured for np-Au, reprinted from [89] with permission of Elsevier; (**b**) Force-displacement curves measure for TiAl with spherical indentation [143], reproduced with permission of Cambridge University Press.

On the macroscopic level, the stress–strain trajectories shown in Figure 21 show inspiring similarities. The loading history applied in [89] contains inserted unloading–reloading phases, motivated from works on nanoindentation [140,141,142,143]. In these works, it is shown that the hysteresis loops indicate kinematic hardening, which models the repulsion of dislocations that pile up at surfaces or grain boundaries on the microscopic level. Further, we can also expect visible creep if we insert a period of constant load before unloading begins (see insert in Figure 21b). Consequently, there is room for refining the constitutive model for the solid phase toward viscoplasticity with (nonlinear) isotropic and kinematic hardening as far as these phenomena occur in the experiments. 

Finally, at about 10–20% compression strain the stress progressively increases, as can be seen in Figure 21a. This is assumed to be caused by the formation of contacts between ligaments and compaction of pores [112,135]. For an np-Au with polymer coating, it is demonstrated that contacts formed by the polymer coating can increase the macroscopic Young’s modulus by one order of magnitude [72]. While contact is always correctly included in MD simulations, the literature on FEM so far does not account for internal contacts. This can be attributed to the excessive computation time in case of FE solid models. Large elastic–plastic deformations along with the nonlinearities caused by the numerous potential contacts within the sample immensely slow down the numerical simulation. In the case of FE beam models, it is yet not possible to include the contact between ligaments due to the absence of a contact implementation for the elastic–plastic beam elements in space. Thus, plenty of challenges still exist that provide room for further work in the micromechanical modeling of nanoporous metals.

## 4. Summary and Future Prospects

The previous discussion illustrates that a micromechanical approach purely based on modeling does not exist. Impressive progress has been made by a close interaction of cutting-edge experimental works, inspiring modeling groups to look into the questions raised with the help of computational tools. In many works with highly original and important contributions to the field, both disciplines are strongly interwoven [15,18,19,89,111,112,115,123,135]. Together with these works, where this interaction results from a continuous long-term progression spanning over several groups in the field, an area is formed which deserves to be named experimentally informed micromechanics. Theoretical works inspire experimentalists, as it was the case for the tension–compression asymmetry predicted in [112]. This effect was experimentally confirmed many years later by Lührs et al. [135]. Furthermore, iterative loops form, as the case for the investigation on topological effects in several papers, such as [14,65,80,85], where we look forward to the future development that may hopefully solve this fascinating puzzle about the exponents. 

**Figure 22:**
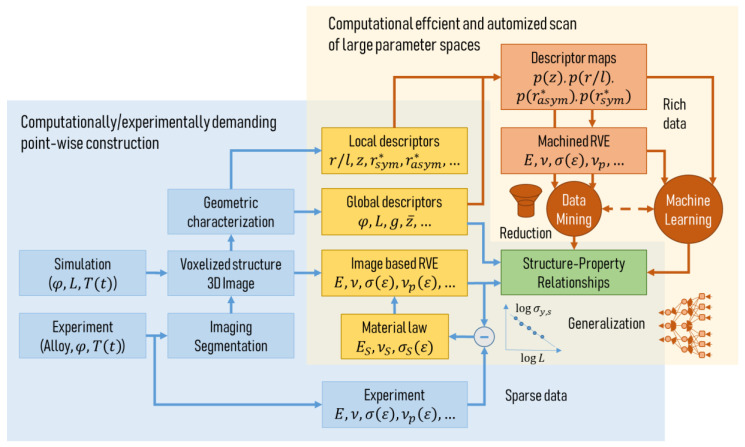
Workflow of the microstructure characterization and micromechanical modeling chain for data generation and identification of the structure–properties relationship of nanoporous metals.

Our view of the development till date and the arising future perspectives is presented in Figure 22, where the first area is highlighted in blue and the latter red. As a common theme, the set of descriptors for geometrical and mechanical properties is continuously extended and improved in its accuracy through the progress made in the various characterization techniques, image processing software, and modeling methods, presented in Section 2 and Section 3. Owing to the rapidly increasing resolution, the progress in software development pushed by open source projects and the increase in computational power combined with efficient modeling approaches, we are moving from sparse data to rich data. This allows for the application of novel approaches, such as data reduction, data mining, and machine learning, that support us in our efforts of decoding and generalization of the underlying structure–properties relationship. 

Depending on the specific goal, there exist elegant ways of taking a short cut from the experiment to the material properties, as indicated in the lower left part of Figure 22. One example to be mentioned in this context is the work of Zabihzadeh et al. [35]. Samples of nanoporous polycrystalline silver with a porosity of 25–30% and pore sizes in the 200–400 nm range were loaded in tension in situ while observing the sample with a high-energy X-ray beam. After tomographic reconstruction of the microstructure, the deformation behavior of the sample was predicted in an FE simulation and the material law was fitted to the experimentally measured macroscopic stress–strain curve. With this approach, the material behavior of the solid fraction can be obtained in a very robust way. A comparison of the evolution of porosity and local strains measured in situ and from the FE simulation showed a very good agreement for all samples. In addition, having simultaneously global and local information from the experiment, see for example [144], can be very helpful, while the common uncertainties due to error propagation along complex characterization and modeling paths are eliminated. It can be expected that such approaches will serve in the future also for complementing the strategies that will be discussed in the following paragraphs. To transfer this technique to nanoporous metals, the resolution in X-ray imaging needs to be improved to reach a few nm. So far, Larsson et al. have achieved a resolution of 62 nm [55]. For samples with smaller ligament sizes, the existing alternatives FIB-SEM tomography [18,19,34] and TEM tomography [16,26] are much more labor-intensive. 

At the same time, the algorithms for image processing need to be further improved, as shown in [50,53]. Currently, valuable information on the morphology is lost or biased due to the nature of the algorithms, causing significant over- or underpredictions by the micromechanical models based on such data. A propagation of such effects can be avoided by using full 3D models in the form of FE solid or FE voxel models, which are, however, computationally very demanding. Independent of the modeling approach, the derived geometric descriptors, serving as independent parameters for the structure–properties relationships, contain still a considerable degree of uncertainty. Further, the flexibility in setting up microstructures with variable morphology and topology using spinodal decomposition [15] or Gaussian random fields [65] is limited. For a deeper investigation of general parameter spaces, modeling techniques require one to choose both types of descriptors independently. This can, for example, be achieved by combining the approaches of [80] and [53] for defining the connectivity and the shape of individual ligaments, respectively. Furthermore, the generation of the structure needs to be expanded by the incorporation of further descriptors. RVEs based on periodic cells, such as Gibson–Ashby, gyroid, or diamond, allow only for limited integration of randomness by perturbation or random deletion. 

In this direction, the first maps of ligament shape distributions already exist [50,91]. Probability densities of the ligament aspect ratio p(r/l), shape $p\left(r_{sym}^{*}\right)$, $p\left(r_{asym}^{*}\right)$, coordination p(z), and more characteristics could be used for producing a large number of artificial structures that are as close to real samples as far as the descriptors are established and analyzed. This will move artificially generated microstructures closer to the real probability distributions as a requirement for the future work that is highlighted with the reddish color in the upper right corner of Figure 22. Having such a tool in our hands, we will be able to efficiently scan large multidimensional parameter spaces of descriptors and reliably predict the macroscopic mechanical properties for any assumed constitutive law on the level of the single ligament, including effects such as surface energy or actuation by charge injection. We will, then, probably move further from rich data to even big data, allowing for mining and decoding of the fundamental structure–properties relationships of nanoporous metals. 

It has been demonstrated in [80] that once we are able to analyze a bigger dataset, we are better able to generalize the gained understanding that can be casted into a simple model. Combining this approach with pioneering simulations on multiaxial loading conditions [123] offers exciting perspectives toward multi-scale simulation schemes that would allow a prediction of the anisotropic deformation behavior with distortion of the yield surface for radial and non-radial loading scenarios. 

In view of the new possibilities of machine learning that are, nowadays, readily available and rapidly entering the field of materials science, we shift from computational modeling based on physical equations toward computer approximation of large datasets that ideally integrate experimental as well as machined data [145,146]. This should, however, not be the end. The goal for which we should strive is to utilize the derived approximations to support our understanding of the physics. Once we reach this level, such novel techniques turn into standard computer methods complementing MD and FE simulations. To this end, by combining high complexity with fascinating properties, nanoporous metals represent a perfect candidate providing a vast amount of challenges which need to be addressed by developments in methods, hardware, and software. In return, experimentally informed micromechanics helps to solve the fundamental questions and deepen the knowledge with the help of micromechanically informed experiments. 

## Figures and Tables

**Table 1:** Software packages applied for the characterization of nanoporous metals. Application types are indicated as 3D volume/interfacial surface (VS), skeleton centerline (SC), feature thickness (FT), and morphological analysis and descriptors (MA). Right column: Selected papers where the software packages have been applied.

Software	OSS	Reference	VS	SC	FT	MA	Applied by
Amira-Avizo	no	ThermoFisher Scientific	X	X	X	X	[35,42,43]
Aquami	yes	[44]	X	X	X	X	[45]
CHomP	yes	[46]	–	–	–	X	[47]
Fiji/ImageJ	yes	[48]	X	X	X	X	[19,42,44,49,50]
iso2meshtoolbox	yes	[51]	X	–	–	–	[18]
Mathematica	no	Wolfram Research	X	X	X	X	[42]
MATLAB	no	MathWorks	X	X	X	X	[34]
MAVI	no	Fraunhofer ITWM	X	X	X	X	[16]
OVITO	yes	[52]	X	–	–	–	[53]
Pore3D	yes	[54]	–	X	X	–	[55]

## Note

Image reprint permissions for Figure 1: From left to right and top to bottom, the figures are reprinted from: Li and Sieradzki (with permission from [25] Copyright (2020) by the American Physical Society; Rösner et al. [16] with permission from John Wiley and Sons; Fujita et al. [26] with the permission of AIP Publishing; Chen et al. [17] with the permission of AIP Publishing; Saane et al. [15] with permission from Elsevier; Mangipudi et al. [34] with permission from Elsevier; Ziehmer et al. [42] with permission from Elsevier; Fujita et al. [26] with the permission of AIP Publishing; Mangipudi et al. [18] with permission from Elsevier; Pia et al. [91] with permission from Elsevier, and Biener et al. [10] with the permission of AIP Publishing; with permission from Hu [36]; Stuckner et al. [44] with permission from Elsevier; Ziehmer et al. [42] with permission from Elsevier; Mangipudi et al. [18] with permission from Elsevier; Li et al. [47] available under the terms of the Creative Commons Attribution 4.0 International license (CC BY 4.0); Fujita et al. [26] with the permission of AIP Publishing; Rösner et al. [16] with permission from John Wiley and Sons; Fujita et al. [26] with the permission of AIP Publishing; Chen et al. [17] with the permission of AIP Publishing; with permission from Hu [36]; Liu et al. [78] with permission from Elsevier; Pia et al. [91] with permission from Elsevier; Richert and Huber, own work [50] distributed under the Creative Commons Attribution 4.0 International license (CC BY 4.0). Image reprint permissions for Figure 11: From bottom to top and left to right, these images are reprinted from: Liu and Antoniou [93] with permission from Elsevier; Huber et al. [89] under Attribution-NonCommercial-NoDerivs 3.0 Unported license (CC BY-NC-ND 3.0), own work; Farkas et al. [112] with permission from Elsevier; Sun et al. [117] with the permission of AIP Publishing; Feng et al. [129] with the permission of AIP Publishing; Lührs et al. [135] according to ACS AuthorChoice License, own work; Saane et al. [15], with permission from Elsevier; Sieradzki et al. [139] adapted with permission from Sieradzki et al. copyrighted by the American Physical Society; Huber [80] published under Creative Commons Attribution License (CC BY 4.0), own work; Richert et al. [53] published under Creative Commons Attribution License (CC BY 4.0), own work; Mangipudi et al. [18], with permission from Elsevier; Hu et al. (2016) with permission from Elsevier and by courtesy of K. Hu [36]; Soyarslan et al. [65], with permission from Elsevier.

## Abbreviations

BS	biggest-sphere
DART	discrete algebraic reconstruction
DFT	density functional theory
ECD	electrochemical dealloying
EDT	Euclidean distance transform
ET	electron tomography
FE	finite element
FEM	finite element method
FIB	focused ion beam
GA	Gibson–Ashby
IND	interfacial normal distributions
ISD	interfacial shape distribution
KMC	kinetic Monte Carlo
LMD	liquid metal dealloying
MAT	medial axis transform
MD	molecular dynamics
NPG	nanoporous gold
OSS	open-source software
PCF	pair correlation function
PXCT	ptychographic X-ray computed tomography
ROI	Region of Interest
RVE	representative volume element
SANS	small-angle neutron scattering
SEM	scanning electron microscopy
STL	standard triangulation language
TEM	transmission electron microscopy
Th	Thickness (biggest-sphere)
TVM	total variation minimization
TXM	transmission X-ray microscopy
VOI	Volume of Interest
WBPJ	weighted back-projection

## Symbols

〈·〉	average of ·
γ	surface energy
ε	strain
ζ	cut fraction
$\zeta_{c}$	percolation threshold (cut fraction)
$\zeta_{tot}$	total cut fraction
$\kappa_{1},\kappa_{2}$	principle curvatures
ν	macroscopic Poisson’s ratio
$\nu_{p}$	plastic Poisson’s ratio
σ	stress
$\sigma_{u}$	ultimate tensile strength
$\sigma_{y}$	macroscopic yield stress
$\sigma_{ys}$	yield stress of the solid phase
φ	solid fraction
$\varphi_{c}$	percolation threshold (solid fraction)
$\varphi_{eff}$	effective solid fraction
a	parabolic function constant (ligament shape)
A	randomization of beam network
b	concavity parameter (ligament shape)
$c_{R}$	size of the nodal mass
$C_{b}$	leading constant GA (bending)
$C_{E}$	leading constant GA (elastic)
$C_{t}$	leading constant GA (tension)
$C_{\sigma }$	leading constant GA (yield)
$C_{V}$	scaled connectivity density
d	ligament diameter (thickness)
E	macroscopic Young’s modulus
$E_{T}$	work hardening rate
$E_{s}$	Young’s modulus of the solid phase
$E_{s}$	surface elastic modulus
f	percolation exponent
$f_{0}$	residual surface stress
G	genus
g	scaled genus
H	hardness
$K_{IC}$	fracture toughness
l	ligament length
L	characteristic length scale (feature)
$\tilde{L}$	characteristic spacing
$L_{RVE}$	RVE edge length
n	exponent coarsening power law
$n_{E}$	exponent GA equation (elastic)
$n_{\sigma }$	exponent GA equation (yield)
p	porosity
$p_{c}$	percolation threshold (porosity)
$q_{a}$	charge density
r	ligament radius
$r_{sym}^{*}$	ligament shape parameter (symmetry)
$r_{asym}^{*}$	ligament shape parameter (asymmetry)
r/l	ligament aspect ratio
$S_{V}$	surface area per volume
$S_{V}^{-1}$	inverse specific surface area
t	coarsening time
V	volume
$V_{s}/A_{s}$	volume-to-surface ratio
z	coordination number (local)
$\bar{z}$	average coordination number
